# Meta‐analysis of salt marsh vegetation impacts and recovery: a synthesis following the *Deepwater Horizon* oil spill

**DOI:** 10.1002/eap.2489

**Published:** 2021-12-08

**Authors:** Scott Zengel, Jennifer Weaver, Irving A. Mendelssohn, Sean A. Graham, Qianxin Lin, Mark W. Hester, Jonathan M. Willis, Brian R. Silliman, John W. Fleeger, Giovanna McClenachan, Nancy N. Rabalais, R. Eugene Turner, A. Randall Hughes, Just Cebrian, Donald R. Deis, Nicolle Rutherford, Brian J. Roberts

**Affiliations:** ^1^ Research Planning, Inc. (RPI) Tallahassee Florida 32303 USA; ^2^ Research Planning, Inc. (RPI) Columbia South Carolina 29201 USA; ^3^ Louisiana State University Baton Rouge Louisiana 70803 USA; ^4^ Gulf South Research Corporation Baton Rouge Louisiana 70820 USA; ^5^ University of Louisiana at Lafayette Lafayette Louisiana 70504 USA; ^6^ Nicholls State University Thibodaux Louisiana 70301 USA; ^7^ Duke University Marine Laboratory Beaufort North Carolina 28516 USA; ^8^ Louisiana Universities Marine Consortium Chauvin Louisiana 70344 USA; ^9^ Northeastern University Marine Science Center Nahant Massachusetts 01908 USA; ^10^ Northern Gulf Institute Stennis Space Center Mississippi State University Starkville Mississippi 39529 USA; ^11^ Atkins Sciences Jacksonville Florida 32256 USA; ^12^ National Oceanographic and Atmospheric Administration (NOAA) Seattle Washington 98115 USA

**Keywords:** coastal wetland, *Deepwater Horizon*, ecological disturbance, ecological impact, ecological recovery, ecological restoration, Gulf of Mexico, *Juncus roemerianus*, natural resource damage assessment, oil spill, salt marsh, *Spartina alterniflora*

## Abstract

Marine oil spills continue to be a global issue, heightened by spill events such as the 2010 *Deepwater Horizon* spill in the Gulf of Mexico, the largest marine oil spill in US waters and among the largest worldwide, affecting over 1,000 km of sensitive wetland shorelines, primarily salt marshes supporting numerous ecosystem functions. To synthesize the effects of the oil spill on foundational vegetation species in the salt marsh ecosystem, *Spartina alterniflora* and *Juncus roemerianus*, we performed a meta‐analysis using data from 10 studies and 255 sampling sites over seven years post‐spill. We examined the hypotheses that the oil spill reduced plant cover, stem density, vegetation height, aboveground biomass, and belowground biomass, and tracked the degree of effects temporally to estimate recovery time frames. All plant metrics indicated impacts from oiling, with 20–100% maximum reductions depending on oiling level and marsh zone. Peak reductions of ~70–90% in total plant cover, total aboveground biomass, and belowground biomass were observed for heavily oiled sites at the marsh edge. Both *Spartina* and *Juncus* were impacted, with *Juncus* affected to a greater degree. Most plant metrics had recovery time frames of three years or longer, including multiple metrics with incomplete recovery over the duration of our data, at least seven years post‐spill. Belowground biomass was particularly concerning, because it declined over time in contrast with recovery trends in most aboveground metrics, serving as a strong indicator of ongoing impact, limited recovery, and impaired resilience. We conclude that the *Deepwater Horizon* spill had multiyear impacts on salt marsh vegetation, with full recovery likely to exceed 10 years, particularly in heavily oiled marshes, where erosion may preclude full recovery. Vegetation impacts and delayed recovery is likely to have exerted substantial influences on ecosystem processes and associated species, especially along heavily oiled shorelines. Our synthesis affords a greater understanding of ecosystem impacts and recovery following the *Deepwater Horizon* oil spill, and informs environmental impact analysis, contingency planning, emergency response, damage assessment, and restoration efforts related to oil spills.

## Introduction

Marine oil spills continue to be a global issue, with coastal wetlands, including salt marshes, among the most sensitive habitats at risk during spills. These habitats support numerous ecosystem functions and services, making oil spill impacts to coastal wetlands particularly important from an ecological and human perspective (Farrington 2013, 2014). The 2010 *Deepwater Horizon* oil spill was the largest marine oil spill in US waters to date and among the largest worldwide. Approximately 4.1 M barrels (~560,000 metric tons) of South Louisiana crude oil, a medium‐weight crude, were released into the Gulf of Mexico (McNutt et al. 2012), and at least 2,113 km of coastal shorelines were oiled (Nixon et al. 2016). Coastal wetlands accounted for at least 1,105 km (52%) of oiled shorelines, 95% of which were in the Mississippi Delta Region of Louisiana (Nixon et al. 2016), where large expanses of coastal salt marsh provide numerous ecosystem services and functions, including coastal protection, fish and wildlife habitat provisioning, water quality maintenance, commercial and recreational fisheries production, and carbon sequestration (Mendelssohn et al. 2012, Mitsch et al. 2015). *Spartina alterniflora* and *Juncus roemerianus* serve as the foundation species for salt marshes in the region, providing physical structure, primary productivity, habitat cover, organic matter and detritus, food web support, nutrient cycling, and the development and stabilization of marsh soils, thereby greatly influencing ecosystem processes and associated species in the marsh and surrounding estuary (Engle 2011, Mendelssohn et al. 2012). For these reasons, it is valuable to synthesize our understanding of salt marsh vegetation impacts and recovery following this spill of national and international significance.

Several publications have addressed impacts of the *Deepwater Horizon* oil spill on salt marsh vegetation, either directly focused on the vegetation or while studying associated species or processes (Lin and Mendelssohn 2012, Silliman et al. 2012, Anderson and Hess 2012, McClenachan et al. 2013, Zengel et al. 2015, 2016*a*, 2021, Lin et al. 2016, Hester et al. 2016, Willis et al. 2016, Silliman et al. 2016, Fleeger et al. 2018, 2019, Zerebecki et al. 2021). The initial impacts of this oil spill on salt marsh vegetation have been found to be mostly consistent across studies, particularly for heavily oiled marshes, although fewer studies have reported on marshes with moderate oiling and especially with lighter oiling. Reported impacts include: (1) vegetation dieback and denuded shorelines; (2) reduced aboveground vegetation cover, stem density, plant height, biomass, and vegetation condition; and (3) reduced belowground biomass and soil strength leading to increased marsh erosion. In contrast, vegetation recovery has been variable among studies, ranging from a few years to incomplete recovery over several years. These findings on *Deepwater Horizon* impacts and recovery are generally consistent with studies from other oil spills, particularly larger spills involving widespread heavy and persistent oiling, although each major spill also has unique characteristics (Mendelssohn et al. 2012, Michel and Rutherford 2014, Pezeshki and DeLaune 2015).

Each of the *Deepwater Horizon* salt marsh vegetation studies mentioned above differed in timing, duration, number of sites, geographic scope, and specific locations and conditions examined relative to salt marsh vegetation attributes and oiling quantities and characteristics. Because natural populations, environmental settings, and oiling conditions can be quite variable in space and time, individual studies with limited geographic or temporal resolution may not completely describe ecological effects after large oil spills, therefore there is a need for integration of data across multiple studies (Fodrie et al. 2014). Here, we use meta‐analysis to synthesize published and unpublished data from 10 studies and 255 sampling sites across Louisiana, Mississippi, and Alabama over seven years post‐spill (2010–2017), including analyses of multiple Gulf of Mexico Research Initiative (GoMRI) and Natural Resources Damage Assessment (NRDA) datasets. We tested the hypotheses that the oil spill reduced plant cover, stem density, vegetation height, aboveground biomass, and belowground biomass compared with reference marshes. We evaluated these vegetation metrics individually for *Spartina alterniflora*, *Juncus roemerianus*, and for the total plant community (all species combined, including species such as *Distichlis spicata* and *Spartina patens* when present). We further tracked the degree of effects over time for the different vegetation metrics to assess impact duration and recovery time frames. Our resulting synthesis provides further understanding of impacts arising from the *Deepwater Horizon* oil spill and informs environmental impact analysis, contingency planning, emergency response, damage assessment, and restoration efforts to curb negative impacts from future spills. Our synthesis on impact and recovery time frames should be particularly valuable for environmental analyses of oil exploration, production, and transportation activities, as well as for NRDA injury assessments and restoration planning, where impact‐recovery trajectories are important inputs to Habitat Equivalency Analysis (HEA), Resource Equivalency Analysis (REA), and other similar approaches used for restoration scaling (i.e., determining the type and amount of restoration needed to offset resource impacts; Peterson et al. 2008, Jones and DiPinto 2018, Baker et al. 2020, Fricano et al. 2020).

## Methods

### Studies and data sources

Our analyses included published and unpublished salt marsh vegetation data comparing oiled and reference sites collected after the spill (April 2010), including large GoMRI and NRDA datasets. Data sources included Lin and Mendelssohn (2012), Silliman et al. (2012), McClenachan et al. (2013), Zengel et al. (2015, 2016*a*, 2021), Lin et al. (2016), Hester et al. (2016), Willis et al. (2016), and Zerebecki et al. (2021), plus unpublished data from these studies as well as from co‐authors Rabalais, Hughes, and Cebrian (Appendix S1: Table S1). Detailed methods are explained in each of the published studies and in metadata available through GoMRI’s Information and Data Cooperative (GRIIDC) (https://data.gulfresearchinitiative.org/) and NOAA’s Data Integration Visualization Exploration and Reporting (DIVER) application (https://www.diver.orr.noaa.gov/). The study sites were distributed throughout the northern Gulf of Mexico (Louisiana, Mississippi, and Alabama), with oiled and reference sites well interspersed, and with numerous sites concentrated in southeastern Louisiana, particularly in Barataria and Terrebonne Bays where salt marsh oiling was most widespread and severe (Appendix S1: Fig. S1). We included only ground‐based field studies and did not consider studies based on laboratory, mesocosm, or remotely sensed data. We excluded studies from our analyses when investigators chose to withhold unpublished data for their own planned publications, or when data were collected landward and outside of the main oiling bands on the shoreline (e.g., McCall and Pennings 2012, Zengel et al. 2016*a* [in part], Hill and Roberts 2017; see “*Marsh zones*” below). We preferentially used data received directly from the original research teams who collected, verified, quality controlled, and voluntarily shared data with us, and downloaded only raw datasets from public sources when instructed to do so by the original researchers. In all cases, the original researchers provided guidance on how to best process and apply their data for the analyses and were invited to be co‐authors. For species‐specific metrics, data were included from all studies in which plant cover for the species averaged at least 5% in corresponding reference sites.

Study sites were categorized as oiled or reference by the original investigators using different means and criteria, but all studies documented the presence of oil in their study sites. Several studies included detailed, site‐specific, ground‐based information on the degree of maximum oiling in their sites tied to oiling conditions in 2010–2011, based on Shoreline Cleanup Assessment Technique (SCAT)‐like methods or similar criteria such as oiling band width across the shore, percent cover of oil on the substrate or vegetation, and oiling thickness (see Michel et al. 2013, Nixon et al. 2016), often coupled with oil chemistry data. Five of these studies included groups of sites reliably classified as “heavily oiled” based on the above criteria. Studies with groups of sites reliably classified as “lightly oiled” (one study) or “moderately oiled” (three studies) were limited in number and duration of data. Studies with sites that could not be reliably classified according to maximum oiling degree, or were not replicated by oiling degree, included sites that qualitatively ranged from very lightly to heavily oiled based on their locations and an overview of summary geospatial shoreline oiling data from the SCAT and NRDA programs (Nixon et al. 2016). Therefore, two categories of marsh oiling were subsequently examined in our analyses: “all oiled” sites, which combined the various oiling degrees from unspecified to very light to heavy oiling, versus reference sites (255 sites total); and confirmed heavily oiled sites versus reference sites (130 sites total). We decided to use the all oiled category so that we could incorporate as many existing studies, datasets, and sites as possible, and so that a range of oiling levels that were not strictly heavily oiled could be examined. The heavily oiled sites were of interest to enable investigation of more intense impacts and subsequent recovery. Data analyses from studies with groups of sites reliably classified as lightly oiled and moderately oiled are included as Supporting Information with limited interpretation given their small sample size (see Appendix S2: Figs. S1–S5, Table S1; these data were also included in the all oiled sites as part of the main analyses). We did not compare statistically the all oiled sites to the heavily oiled sites because they were not independent groups (i.e., the all oiled sites included the heavily oiled sites).

Some oiled sites in our study had active shoreline cleanup treatments applied as part of the *Deepwater Horizon* emergency response. Cleanup treatments included manual and mechanical removal of oiled wrack, raking and cutting of oiled and dead marsh vegetation, and raking and scraping of oil deposits from the marsh substrate (Zengel and Michel 2013, Zengel et al. 2015). Approximately 19% of the all oiled sites and 39% of the heavily oiled sites were known to have had active shoreline cleanup treatments (based on Zengel and Michel 2013, and details from individual investigators and studies). All or nearly all the oiled sites may have had passive treatment involving sorbent boom deployment just seaward of the marsh edge, often followed by boom stranding in the marsh and subsequent retrieval operations (Zengel and Michel 2013). Some oiled sites may also have had low pressure ambient water flushing or other treatments. Some studies did not specify whether their sites were actively treated. Therefore, we decided to pool all the oiled sites regardless of whether they were actively treated or not. In this regard, our analysis is somewhat unique in that it examines the overall impacts of the oil spill, including effects from both oiling and associated shoreline cleanup treatments, although these were not differentiated. In contrast, most of the prominent vegetation studies published to date focused on oiled sites that specifically did not have cleanup treatments (Lin and Mendelssohn 2012, Lin et al. 2016, Hester et al. 2016; see Zengel et al. 2015, 2016*a*, 2021 for direct comparisons of oiled sites with and without cleanup treatments). Data for oiled sites with shoreline stabilization or restoration treatments that included vegetation planting were limited and were not included in the analyses.

Nearly all sites, whether reference or oiled, were in mainland herbaceous salt marshes with muddy and moderately organic soils. Several sites were in back barrier island salt marsh and may have had somewhat sandier soils. The salt marsh vegetation in our study sites was dominated by *Spartina alterniflora* and in some cases was co‐dominated, and in fewer cases dominated, by *Juncus roemerianus*. We excluded data from mangrove‐dominated and mixed marsh and mangrove habitats (i.e., habitats dominated or co‐dominated by *Avicennia germinans*), although we did not exclude salt marsh sites that included scattered mangrove shrubs. Reference versus oiled sites within studies were compared by their respective investigators across metrics such as soils, salinity, inundation, vegetation types, etc., and were found to be similar in terms of habitat characteristics, other than oiling conditions and subsequent impacts to the marsh habitat.

### Marsh zones

Oil was deposited to the greatest degree along the seaward edge of marshes, with gross visible oiling typically limited to ~10–20 m from the shoreline, although oiling extended further into the marsh in some areas (Lin and Mendelssohn 2012, Silliman et al. 2012, Kokaly et al. 2013, Michel et al. 2013, Turner et al. 2014, Zengel et al. 2015). Oiling levels, vegetation and soil characteristics, and marsh functional attributes vary between the marsh edge and interior (Peterson and Turner 1994, Peterson et al. 2008, Hester et al. 2016, Zengel et al. 2016*a*, Rouhani et al. 2017); therefore, we divided the data into two “marsh zones” (after Zengel et al. 2016*b*, 2017). The first zone was defined as the seaward “oiled marsh edge,” where oiling was typically heaviest (~0–6 m from the shoreline, depending on oiling width across shore). A second zone was defined as the “oiled marsh interior” within the main oiling band (~6–20 m from the shoreline, depending on oiling width). Because of shoreline erosion, sampling position relative to distance from the shoreline at the onset of data collection was not static over time across all studies (some studies dropped sites as they eroded, others allowed sites to recede with the shoreline). However, even in later sampling years, oiled sites were still located within areas thought to have been originally affected by oiling, although differences in marsh oiling zones with time may have become less distinct. In all cases, zones in the oiled and reference sites were located at similar distances from the shorelines at the time of sampling.

### Vegetation metrics

#### Plant cover

Most studies (90%) estimated plant percent cover (%) by species and in total using 0.25–1 m^2^ quadrats or larger plots (e.g., 15–50 m^2^ plots) (Appendix S1: Table S1). Most studies reported live cover values, but some did not specifically differentiate live and standing dead (or senescent) plant cover. For studies that sampled across multiple seasons, we focused on data collected in summer and early fall, to correspond with the peak of the growing season (applies to all metrics). Data were compiled for 35 study‐zone‐by‐year combinations for *Spartina alterniflora* cover, 13 study‐zone‐by‐year combinations for *Juncus roemerianus* cover, and 37 study‐zone‐by‐year combinations for total plant cover (all species combined).

#### Stem density

A subset of studies (50%) estimated live plant stem density using quadrats (no. m^−2^) (Appendix S1: Table S1). Data were compiled for 25 study‐zone‐by‐year combinations for *Spartina alterniflora* stem density, 13 study‐zone‐by‐year combinations for *Juncus roemerianus* stem density, and 25 study‐zone‐by‐year combinations for total stem density (all species combined).

#### Vegetation height

A subset of studies (60%) examined vegetation height (cm) (Appendix S1: Table S1). The specific measurements varied somewhat between studies but included variables such as canopy height and maximum or average plant stem or leaf length for the dominant plant species. Data were compiled for 24 study‐zone‐by‐year combinations for vegetation height, not species‐specific.

#### Aboveground biomass

A subset of studies (40%) estimated live aboveground plant biomass using quadrat clippings, reported as areal dry biomass (g m^−2^) (Appendix S1: Table S1). Data were compiled for 23 study‐zone‐by‐year combinations for *Spartina alterniflora* aboveground biomass, 13 study‐zone‐by‐year combinations for *Juncus roemerianus* aboveground biomass, and 23 study‐zone‐by‐year combinations for total aboveground biomass (all species combined).

#### Belowground biomass

A subset of studies (30%) estimated belowground plant biomass using shallow (≤30 cm) cores (Appendix S1: Table S1). Studies varied in either reporting live belowground biomass or total (live and dead) belowground biomass, reported as dry biomass (g m^−2^). Data were compiled for 18 study‐zone‐by‐year combinations for belowground biomass, not specific to species.

### Analysis

We examined oil spill impacts and recovery for each vegetation metric with effect size response ratios, using the natural log (ln) of the ratio of each mean metric value at oiled sites to the corresponding mean metric value at reference sites for each study‐zone‐by‐year combination (after Hedges et al. 1999, Zengel et al. 2016*b*, 2017). The natural log response ratio (ln[RR]) is zero if oiled and reference sites are identical, and negative if the metric, such as plant cover, was lower at oiled sites. As negative ln(RR) values become larger in magnitude, the degree of effects for oiled sites relative to reference sites is greater. For example, ln(RR) = −0.7 is a ~50% reduction in a metric for oiled sites relative to reference, ln(RR) = −2.3 is a ~90% reduction, and ln(RR) = −4.6 is a ~99% reduction. For each metric, we calculated the annual mean ln(RR) (the mean ln[RR] across all contributing sources) and plotted these by year to evaluate impact and recovery by metric and zone. Our interpretation of impact and recovery was based primarily on effect size trends shown in the figures (e.g., mean ln[RR] values dropping below zero after the initial impact and then rising back toward zero over time as recovery progressed). To define recovery, we examined terminal years of individual or grouped ln(RR) values which were zero, approaching zero, or oscillating around zero (e.g., at least one year reaching zero, but preferably two to three years of terminal values at or fluctuating around zero, to define full recovery for a metric). In addition, to confirm our visual interpretations and statistically test for an overall effect (impact) of the spill on each metric over our study period, we compared mean ln(RR) values to zero for all sampling years combined using a random effects model. We report various tabular summaries of the data, including statistical output with both raw *P*‐values and adjusted *P*‐values calculated using the Benjamini–Hochberg (B–H) method (due to the number of comparisons conducted) (see Appendix S1). We generally considered statistical significance as *P* ≤ 0.10; however, based on recent guidance (Wasserstein et al. 2019, Smith 2020), we did not use this or any other value as a dichotomous cutoff point, choosing instead to form our overall interpretations based on the combination of the plotted response ratio (effect size) data, including visual trends and tendencies in the data, in combination with the statistical results. Analyses were conducted in R version 3.5.1, using the *Metafor* package version 2.0‐0 (Viechtbauer 2010) for the random effects modeling.

## Results

### Plant cover

**Fig. 1:**
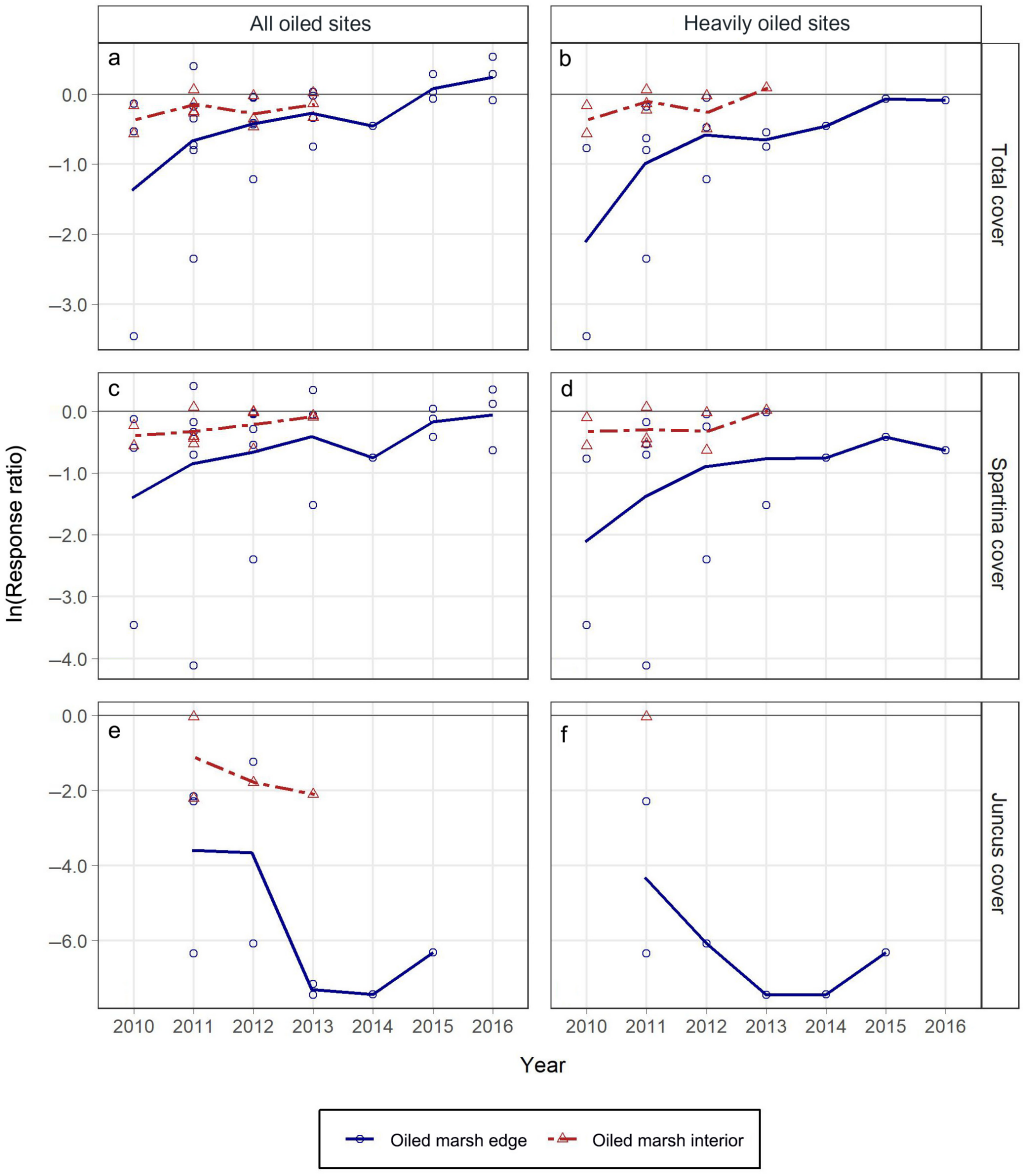
Response ratios (ln[RR]) (oiled/reference) for plant percent cover (%) by marsh zone and year for: (a) total cover for all oiled sites, (b) total cover for heavily oiled sites, (c) *Spartina alterniflora* cover for all oiled sites, (d) *Spartina alterniflora* cover for heavily oiled sites, (e) *Juncus roemerianus* cover for all oiled sites, and (f) *Juncus roemerianus* cover for heavily oiled sites. Lines connect mean annual ln(RR) values by marsh zone across years. ln(RR) values from each contributing source are plotted as open symbols. ln(RR) values less than zero indicate reductions in metrics for oiled sites compared with reference (= impacts). As a guide to interpreting degree of effects, ln(RR) = −0.7 is a ~50% reduction for oiled sites relative to reference, ln(RR) = −2.3 is a ~90% reduction, and ln(RR) = −4.6 is a ~99% reduction.

Total plant cover and *Spartina alterniflora* cover indicated multiyear oil spill impacts, with a maximum 88% mean reduction in cover for the heavily oiled sites at the marsh edge (Fig. 1; Appendix S1: Table S2). Impacts occurred to a greater degree (larger reduction) at the heavily oiled sites than the all oiled sites, and to a greater degree at the marsh edge than in the marsh interior, with general trends toward recovery over time, although full recovery was not observed in all cases. *Juncus roemerianus* cover was more severely impacted (maximum 100% mean reductions at the marsh edge) than *Spartina alterniflora* or total cover, and impacts were ongoing, with no apparent recovery trends. The ln(RR) values across all years combined were lower than zero (indicating impacts) for nearly all plant cover metrics in each oiling category and marsh zone (Appendix S1: Table S3), and when statistical evidence for this was weaker, trajectories indicated probable impacts. Full recovery based on trends through time and the terminal year(s) of ln(RR) values was indicated for total cover (all oiled and heavily oiled sites, both zones) and *Spartina alterniflora* cover for the all oiled sites (both zones) (Fig. 1; Appendix S1: Table S4). Recovery time frames in these cases were generally five to six years post‐spill for the marsh edge and three years post‐spill for the marsh interior. Full recovery was not observed for *Spartina alterniflora* cover in the heavily oiled sites at the marsh edge, or for *Juncus roemerianus* cover in the all oiled sites (both marsh zones) or heavily oiled sites (marsh edge) over the duration of our data records, at least five to six years post‐spill.

### Stem density

**Fig. 2:**
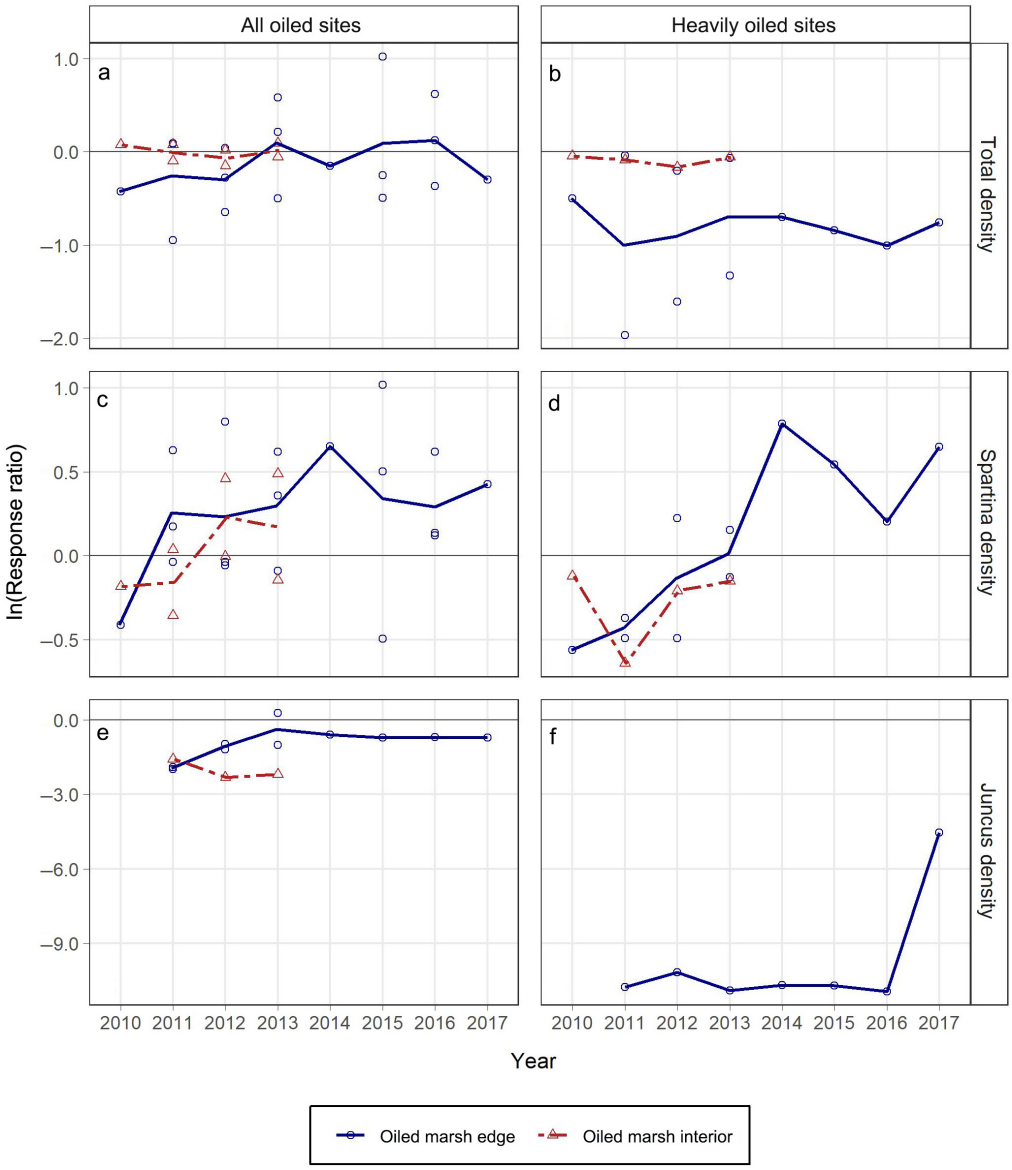
Response ratios (ln[RR]) (oiled/reference) for stem density (no. m^–2^) by marsh zone and year for: (a) total density for all oiled sites, (b) total density for heavily oiled sites, (c) *Spartina alterniflora* density for all oiled sites, (d) *Spartina alterniflora* density for heavily oiled sites, (e) *Juncus roemerianus* density for all oiled sites (note the larger scale on the y‐axis), and (f) *Juncus roemerianus* density for heavily oiled sites. Lines connect mean annual ln(RR) values by marsh zone across years. ln(RR) values from each contributing source are plotted as open symbols. ln(RR) values less than zero indicate reductions in metrics for oiled sites compared with reference (= impacts). As a guide to interpreting degree of effects, ln(RR) = −0.7 is a ~50% reduction for oiled sites relative to reference, ln(RR) = −2.3 is a ~90% reduction, and ln(RR) = −4.6 is a ~99% reduction.

Stem density results were variable, displaying oil spill impacts for some but not all density metrics, with some impacts occurring to a lesser degree and over shorter time periods (Fig. 2; Appendix S1: Table S2). Greater degrees of impact were generally observed for heavily oiled sites at the marsh edge, whereas density metrics were not consistently impacted in the marsh interior. Recovery trends for stem density varied from relatively rapid to a lack of full recovery. *Spartina alterniflora* stem density in the all oiled sites at the marsh edge recovered quickly and displayed an overall increase in stem density across all years combined (Fig. 2; Appendix S1: Table S3). In contrast, impacts on *Juncus roemerianus* stem density were more severe (maximum 86–100% mean reductions) and did not fully recover in the study period. The ln(RR) values across all years combined were lower than zero for four out of 11 cases (Appendix S1: Table S3); however, in four instances for which this was not apparent across all years, trends over time indicated short to medium‐term impacts (e.g., *Spartina alterniflora* density at the marsh edge in the heavily oiled sites over 2010–2013). For metrics that were impacted, full recovery time periods ranged from one to three years to incomplete recovery through at least seven years (Fig. 2; Appendix S1: Table S4). Full recovery was not observed for total stem density in the heavily oiled sites at the marsh edge or for *Juncus roemerianus* stem density in any oil category or zone over the duration of our data records.

### Vegetation height

**Fig. 3:**
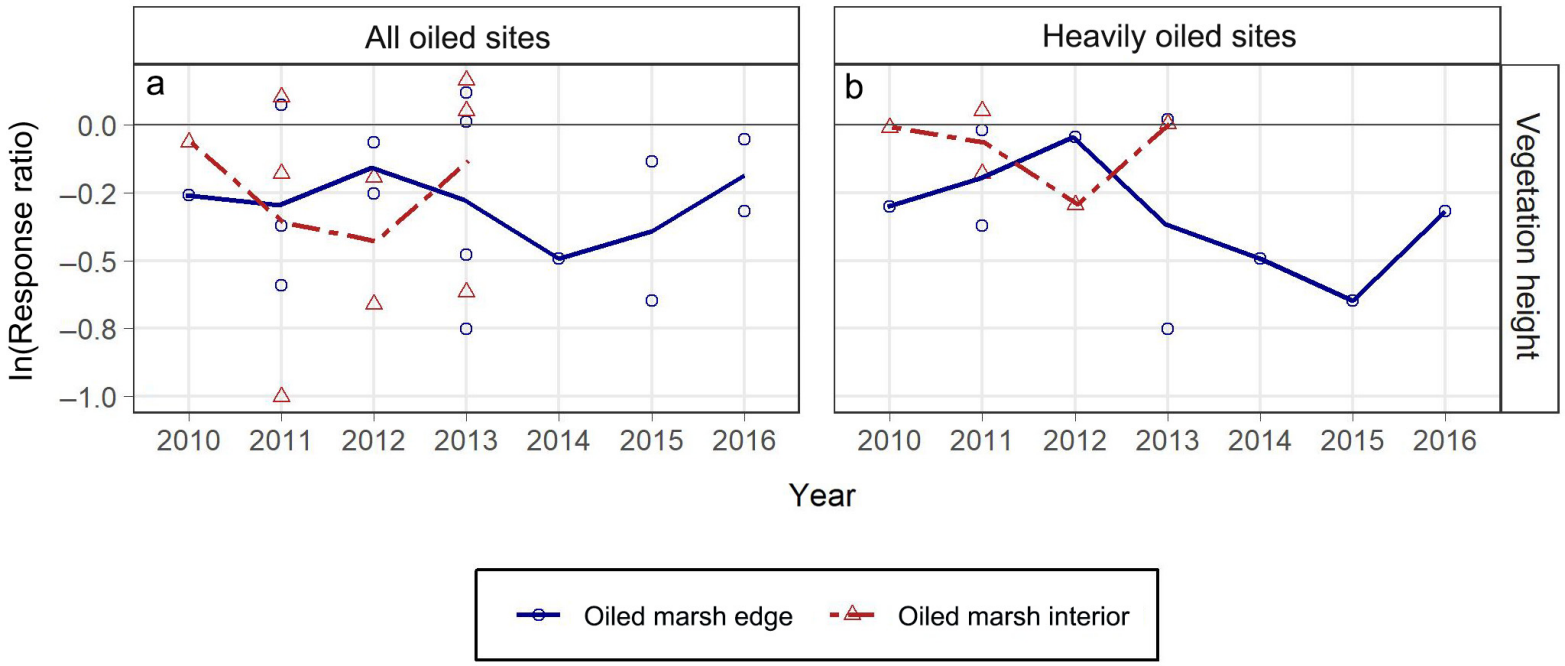
Response ratios (ln[RR]) (oiled/reference) for vegetation height (cm) by marsh zone and year for: (a) all oiled sites, (b) heavily oiled sites. Lines connect mean annual ln(RR) values by marsh zone across years. ln(RR) values from each contributing source are plotted as open symbols. ln(RR) values less than zero indicate reductions in metrics for oiled sites compared with reference (= impacts). As a guide to interpreting degree of effects, ln(RR) = −0.7 is a ~50% reduction for oiled sites relative to reference, ln(RR) = −2.3 is a ~90% reduction, and ln(RR) = −4.6 is a ~99% reduction.

Differences in vegetation height between oiled and reference marshes indicated multiyear oil spill impacts in three out of four cases with general trends toward recovery in later years (Fig. 3). When impacted, the maximum mean reductions in vegetation height ranged from 35 to 48% (Appendix S1: Table S2). The ln(RR) values across all years combined were lower than zero for the all oiled sites at the marsh edge and interior, as well as for the heavily oiled sites at the marsh edge (Appendix S1: Table S3). When impacts were observed, full recovery did not take place over the duration of our data records, at least six years post‐spill for the marsh edge (Fig. 3; Appendix S1: Table S4).

### Aboveground biomass

**Fig. 4:**
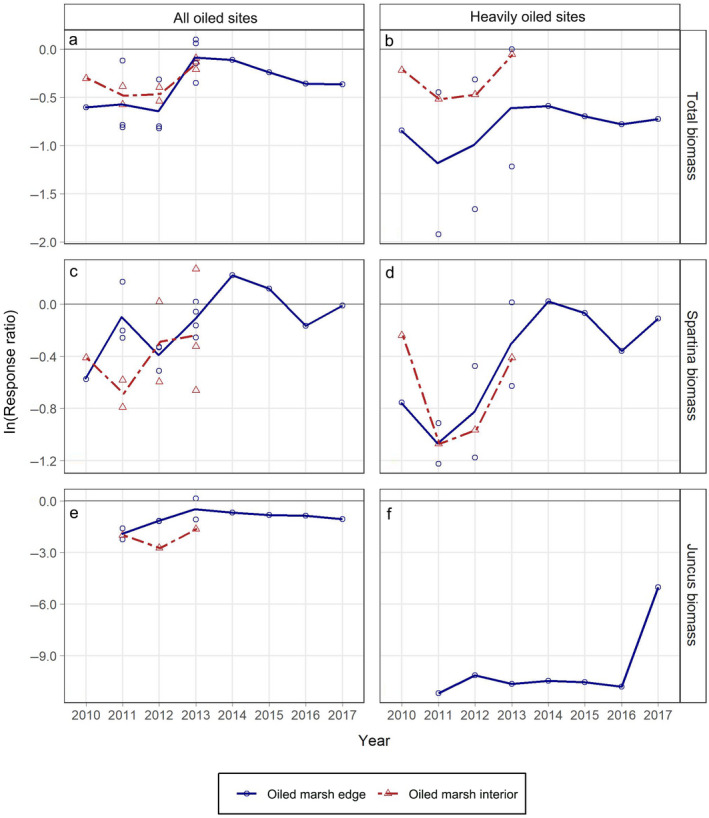
Response ratios (ln[RR]) (oiled/reference) for aboveground biomass (g m^−2^) by marsh zone and year for: (a) total aboveground biomass for all oiled sites, (b) total aboveground biomass for heavily oiled sites, (c) *Spartina alterniflora* aboveground biomass for all oiled sites, (d) *Spartina alterniflora* aboveground biomass for heavily oiled sites, (e) *Juncus roemerianus* aboveground biomass for all oiled sites (note the larger scale on the *y*‐axis), and (f) *Juncus roemerianus* aboveground biomass for heavily oiled sites. Lines connect mean annual ln(RR) values by marsh zone across years. ln(RR) values from each contributing source are plotted as open symbols. ln(RR) values less than zero indicate reductions in metrics for oiled sites compared with reference (= impacts). As a guide to interpreting degree of effects, ln(RR) = −0.7 is a ~50% reduction for oiled sites relative to reference, ln(RR) = −2.3 is a ~90% reduction, and ln(RR) = −4.6 is a ~99% reduction.

Total and *Spartina alterniflora* aboveground biomass indicated multiyear oil spill impacts across zones and oiling categories, with a maximum 69% mean reduction in total aboveground biomass for the heavily oiled sites at the marsh edge (Fig. 4; Appendix S1: Table S2). Impacts occurred to a greater degree for the heavily oiled sites compared with the all oiled sites, and to a similar degree at the marsh edge compared with the marsh interior, except for total biomass at the heavily oiled sites, which showed a greater degree of impact at the marsh edge. General trends toward recovery were observed in most cases, although full recovery had not occurred in several instances (Fig. 4). *Juncus roemerianus* aboveground biomass differed, particularly from *Spartina alterniflora* aboveground biomass, with impacts that were more severe (maximum 100% mean reduction at the heavily oiled marsh edge) and with little recovery. The ln(RR) values across all years combined were lower than zero for nearly all aboveground biomass metrics for both the all oiled sites and the heavily oiled sites for each marsh zone (10 out of 11 cases) (Appendix S1: Table S3). When statistical evidence for this was weaker across all years (one case, *Spartina alterniflora* aboveground biomass in the all oiled sites at the marsh edge), trends over time indicated probable impacts over a shorter time‐period. Full recovery based on trends over time and the terminal years of ln(RR) values were indicated for total aboveground biomass in the heavily oiled sites in the marsh interior, and for *Spartina alterniflora* aboveground biomass for the all oiled sites and the heavily oiled sites at the marsh edge, with recovery times of three to four years post‐spill (Fig. 4; Appendix S1: Table S4). *Spartina alterniflora* aboveground biomass in the marsh interior had not fully recovered through the duration of our data (three years post‐spill) but was tracking similarly to the marsh edge sites where longer term data were available. Full recovery was not observed for total aboveground biomass in the all oiled sites for either marsh zone, for total aboveground biomass in the heavily oiled sites at the marsh edge, or for *Juncus roemerianus* in any instances for at least seven years post‐spill.

### Belowground biomass

**Fig. 5:**
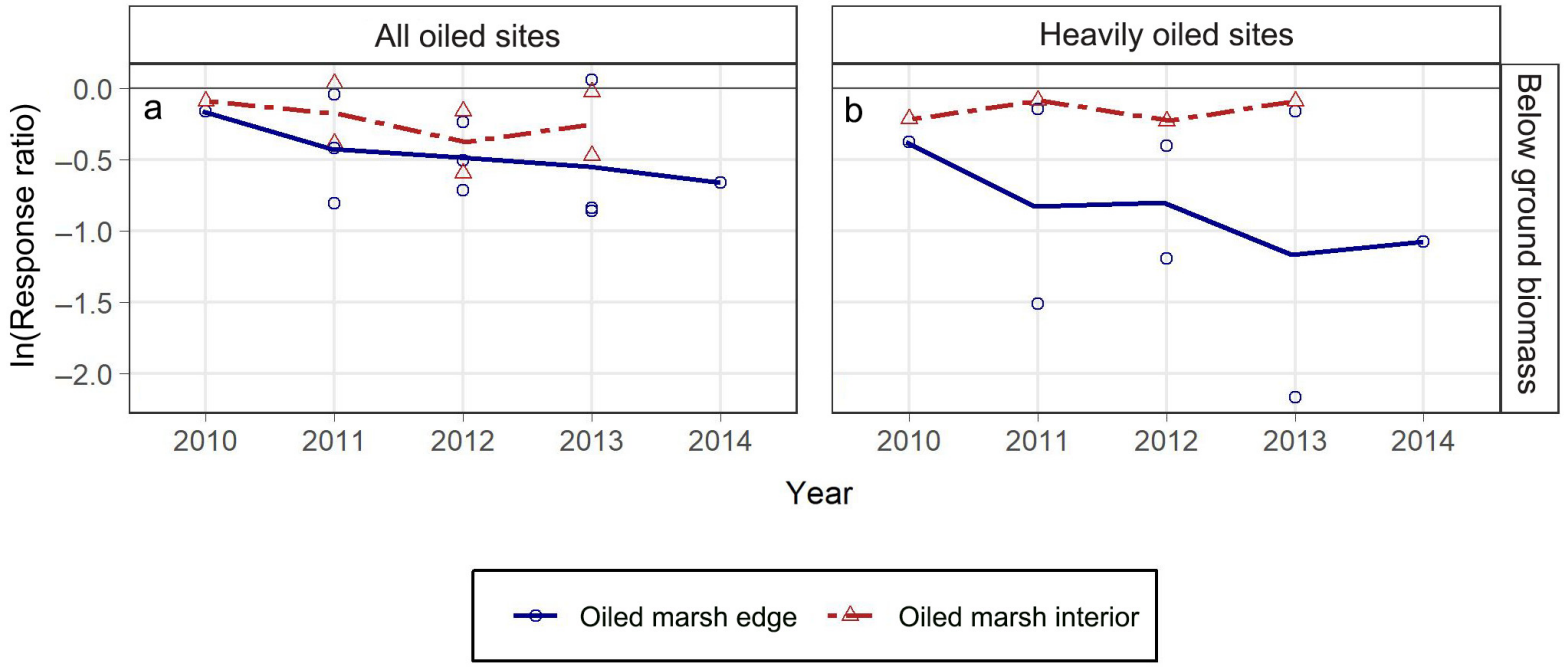
Response ratios (ln[RR]) (oiled/reference) for belowground biomass (g m^−2^) by marsh zone and year for: (a) all oiled sites, (b) heavily oiled sites. Lines connect mean annual ln(RR) values by marsh zone across years. ln(RR) values from each contributing source are plotted as open symbols. ln(RR) values less than zero indicate reductions in metrics for oiled sites compared with reference (= impacts). As a guide to interpreting degree of effects, ln(RR) = −0.7 is a ~50% reduction for oiled sites relative to reference, ln(RR) = −2.3 is a ~90% reduction, and ln(RR) = −4.6 is a ~99% reduction.

Belowground biomass results indicated multiyear oil spill impacts for both oiling categories and all marsh zones with more severe impacts at the marsh edge (maximum 69% mean reduction in the heavily oiled sites), where values were in steady decline over the duration of our observations, in contrast with the recovery trends for many of the other vegetation metrics examined over similar time periods (Fig. 5; Appendix S1: Table S2). The ln(RR) values across all years combined were lower than zero for all belowground biomass metrics (both oiling categories and all zones) (Appendix S1: Table S3). Full recovery was not observed over the duration of our data, through four years for the marsh edge and three years for the marsh interior (Fig. 5; Appendix S1: Table S4). Impacts to belowground biomass were also observed for moderately oiled sites at the marsh edge, with degrees of impact similar to the all oiled sites, including the absence of recovery over the duration of our data, through four years post‐spill (Appendix S2: Fig. S5, Table S1).

## Discussion

### 1 Impacts and recovery

All plant metrics examined indicated impacts from oiling, with 20–100% maximum reductions across oiling categories and marsh zones. In the heavily oiled sites, ~70–90% peak reductions were observed for total plant cover, total aboveground biomass, and belowground biomass at the marsh edge. The steady decline in belowground biomass at the marsh edge over time was particularly concerning for marsh resilience and stability, given the high background rates of shoreline retreat and marsh loss in much of the study area, particularly in southeast Louisiana (Wilson and Allison 2008), and the fact that intact belowground biomass at the marsh edge is a key factor in slowing erosion rates (Silliman et al. 2016, 2019). Belowground biomass impacts and lack of recovery in the moderately oiled sites is also important in this regard and has not been reported previously.

**Fig. 6:**
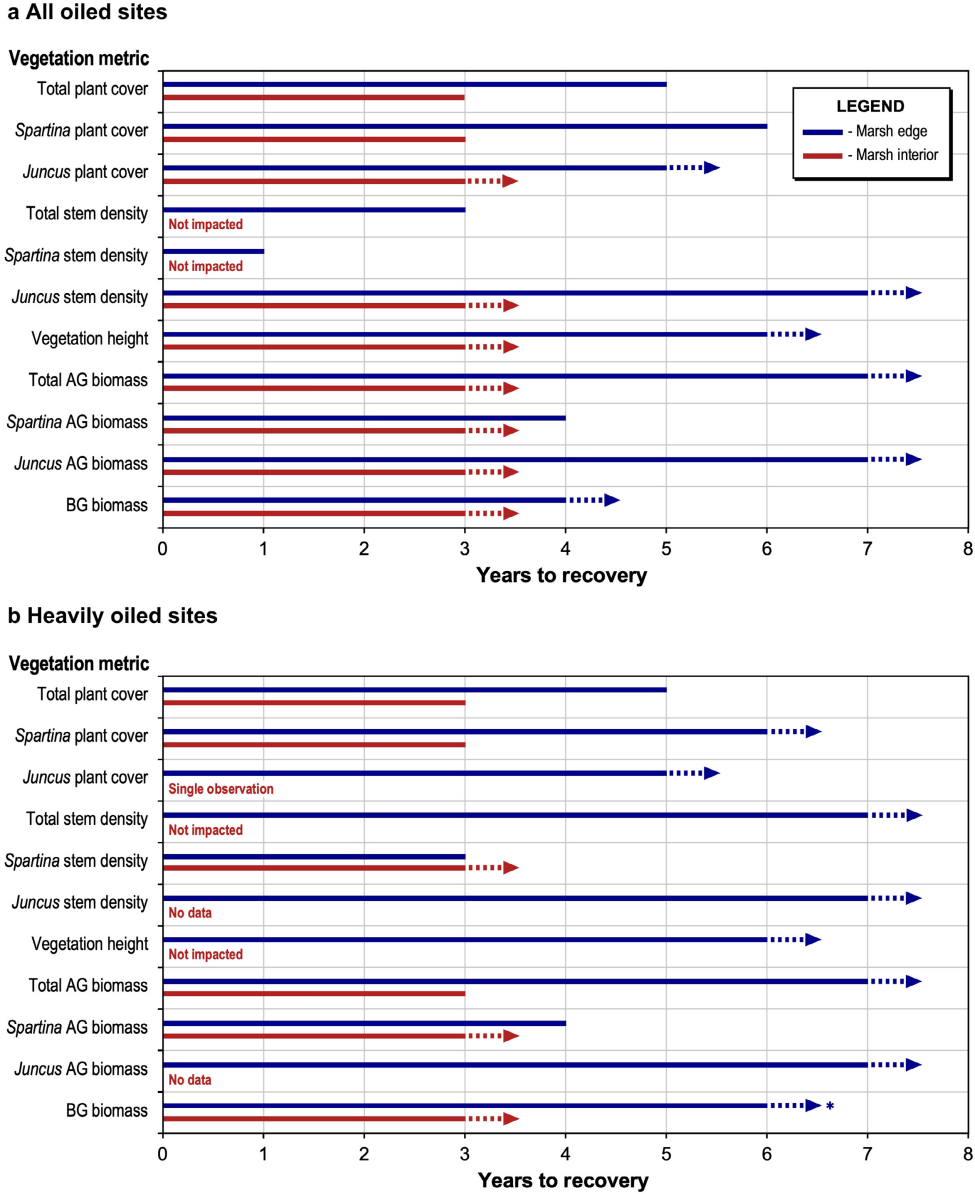
Summary of oiled marsh vegetation recovery time frames for the *Deepwater Horizon* oil spill: (a) all oiled sites, (b) heavily oiled sites. Species are *Spartina alterniflora* and *Juncus roemerianus*. For biomass, AG is aboveground and BG is belowground. Dotted lines and arrows indicate the absence of full recovery through the duration of our study (full recovery indeterminate but exceeding the year indicated). *Includes results from Fleeger et al. (2018) for belowground biomass.

Most plant metrics showed recovery time frames of three years or more, including multiple metrics in which recovery did not occur over the duration of our data, at least seven years post‐spill (summarized in Fig. 6). Total stem density and *Spartina alterniflora* stem density were the main exceptions, lacking impacts in some cases, with lesser impacts and more rapid recovery otherwise, and exceeding reference values in some instances over time. Increased *Spartina alterniflora* stem density may have been stimulated by residual oiling (Lin and Mendelssohn 1998) or unoccupied space and reduced competition due to oiling impacts, including reductions in *Juncus roemerianus* (Lin et al. 2016). However, increased stem density did not contribute to equivalent increases in plant cover or biomass. Investment in stems with lower biomass may indicate less healthy plants and less live leaf material, with potential impacts on overall photosynthesis, habitat provisioning, food web support, and other ecosystem functions.

*Juncus roemerianus* was impacted to a greater degree compared with *Spartina alterniflora* across all metrics, with much slower recovery or lack thereof, consistent with most individual *Deepwater Horizon* studies (Anderson and Hess 2012, Lin and Mendelssohn 2012, Zengel et al. 2015, 2021, Lin et al. 2016, Willis et al. 2016). The exception was Hester et al. (2016), probably because *Juncus roemerianus* was only a minor component of their study sites. A comparative greenhouse experiment with standardized applications of *Deepwater Horizon* oil showed that initial and medium‐term (7 month) vegetation impacts were greater for *Juncus roemerianus* than for *Spartina alterniflora* across multiple metrics, indicating the higher sensitivity of *Juncus roemerianus* to oil exposure (Lin and Mendelssohn 2012). Furthermore and more generally, experimental studies involving plant removal, clonal severing, and transplanting vegetation across flooding and salinity stress gradients in the absence of oil indicated that *Spartina alterniflora* is more tolerant to environmental stress than *Juncus roemerianus*, whereas *Juncus roemerianus* is a stronger competitor than *Spartina alterniflora* (Pennings and Callaway 2000, Pennings et al. 2003, 2005). Based on these studies, in a scenario with an acute stressor or disturbance, *Juncus roemerianus* would be expected to be more severely impacted and recover more slowly, whereas *Spartina alterniflora* would be expected to be less affected, and recover more quickly, with the ability to invade adjacent areas formerly occupied by *Juncus roemerianus*. *Juncus roemerianus* would be expected to eventually return and outcompete *Spartina alterniflora* (or other colonizing and less competitive species) in areas where it was formerly dominant, assuming elevation and soil conditions remained similar; however, the return of *Juncus roemerianus* may take many years (exceeding 10 years in the removal experiments described above; Steven Pennings, pers. commun.). Observations and experiments following the *Deepwater Horizon* spill seem to conform to this model. Had the *Deepwater Horizon* oil spill resulted in widespread heavy oiling in the *Juncus roemerianus*‐dominated marshes of the northeastern Gulf of Mexico (Mississippi, Alabama, and northwestern Florida) (Anderson and Hess 2012, Willis et al. 2016), similar to the heavy oiling levels observed in the *Spartina alterniflora*‐dominated marshes of southeastern Louisiana, vegetation impacts may have been even greater than those observed, and recovery times longer (see Anderson and Hess [2012] for a short‐term mesocosm study examining the impact side of this scenario). Beland et al. (2017) made a similar point based on their remote‐sensing studies and literature review.

The heavily oiled sites in our study generally showed a greater degree of impacts and longer recovery time frames than the all oiled sites, particularly for the marsh edge, although there were cases in which recovery time frames were similar across degrees of oiling. In comparison with the marsh edge, the oiled marsh interior tended to have less severe impacts and more rapid recovery, probably due to less oil reaching the marsh interior in many instances (Silliman et al. 2012, Hester et al. 2016, Rouhani et al. 2016, Zengel et al. 2016*a*). However, impacts were still detected in the marsh interior and recovery in some cases tracked similarly to the marsh edge. Overall, considering the combination of metrics, full vegetation recovery was not observed for either the heavily oiled or the all oiled sites, especially at the marsh edge. The lack of recovery spanned several *Spartina alterniflora* and total vegetation metrics, all marshes with a co‐dominant or dominant *Juncus roemerianus* component, and belowground biomass in general. Overall, our study indicates full marsh vegetation recovery taking longer than seven years after the oil spill (where marsh erosion does not preclude recovery, discussed further below).

Considering recovery time frames across individual studies in comparison with our results, two studies reported vegetation recovery as early as two to three years, not including eroded sites in one case (Silliman et al. 2012) and mostly limited to moderately oiled sites in the other (Lin et al. 2016). Several studies have reported lack of full vegetation recovery through three to five years, in some cases focused on heavily oiled sites (Hester et al. 2016, Lin et al. 2016, Willis et al. 2016, Zengel et al. 2021). Based on these findings, predictions from the literature for marsh vegetation recovery time frames following the *Deepwater Horizon* spill have included: one to two years for light to moderate oiling and three to seven years or more for heavy oiling (Michel and Rutherford 2014); five years or more for heavy oiling (Lin et al. 2016); and two to eight years for a range of light to heavy oiling (Baker et al. 2017). Fleeger et al. (2018) extended the results of the prior studies above, indicating a lack of recovery for belowground biomass in heavily oiled sites through six years. Lin et al. (2020) presented a preview of their latest unpublished findings indicating a lack of full recovery for both total live aboveground biomass and live belowground biomass in heavily oiled sites through nine years post‐spill. Similarly, Zengel and colleagues (pers. commun.) observed an ongoing lack of recovery for *Spartina alterniflora* plant cover in their oiled sites during macroinvertebrate sampling through nine years post‐spill (unpublished field notes from sites in Zengel et al. 2015, 2021). Based on our synthesis coupled with the above information, we extend the projections for full vegetation recovery to 10 years or more post‐spill for heavily oiled sites, realizing that erosion may preclude full recovery in many cases. This places the *Deepwater Horizon* oil spill in a class with only a few other crude oil spills for which marsh recovery time frames approached or exceeded 10 years, including the Gulf War spill in the Arabian Gulf, Saudi Arabia (1991), the *Metula* spill in the Strait of Magellan, Chile (1974), and the *Amoco Cadiz* spill on the coast of Brittany, France (1978) (Gilfillan et al. 1995, Mendelssohn et al. 2012, Michel and Rutherford 2014).

Many of the *Deepwater Horizon* vegetation impacts observed here would include permanent marsh losses and absence of full recovery where spill‐induced vegetation impacts led to accelerated erosion. The effects of oil on marsh erosion via vegetation impacts, and the resulting influences of spill‐caused erosion, as well as background erosion rates, on the interpretation of vegetation recovery are difficult to disentangle and are beyond the scope of this study. The consensus in the literature is that oiling impacts to vegetation accelerated marsh erosion for one to three years after the spill, particularly in heavily oiled sites, as has been reported in several studies associated with the datasets used in our analysis (Silliman et al. 2012, 2016, McClenachan et al. 2013, Zengel et al. 2015, Lin et al. 2016), as well as in other *Deepwater Horizon* marsh erosion studies (Beland et al. 2016, 2017, Rangoonwala et al. 2016, Turner et al. 2016, Khanna et al. 2017, Challenger et al. 2021); however, see Deis et al. (2019) and Challenger et al. (2021) for contrasting results and interpretations. Erosion complicates the assessment of vegetation recovery because studies with fixed sites (~68% of the sites in our analysis) lose the ability to detect impacts as sites erode and are no longer sampled, which can be misinterpreted as vegetation recovery (Hester et al. 2016). Similarly, studies that do not use fixed sites (e.g., where sites shift inland or along shore as shoreline position changes, ~32% of the sites in our analysis) may in some cases be examining progressively lesser oiling conditions year‐by‐year as the oiled marsh erodes, which could also be misinterpreted as recovery. We know from our analysis that although vegetation recovery was observed for certain individual metrics, full recovery was not observed, not even across a single class of metrics. The ongoing declines in belowground plant biomass we observed are especially important in this regard, as intact belowground biomass at the marsh edge is the key plant trait that suppresses erosion and generates the shoreline protection function of marshes (Silliman et al. 2019). Multiple studies contributing to our analysis also reported substantial residual oiling on or in the marsh soils across multiple years in heavily oiled sites at the marsh edge (e.g., Hester et al. 2016, Fleeger et al. 2018, Zengel et al. 2021), indicating that shoreline erosion had not entirely moved through or beyond the heavily oiled zones over the duration of these studies. In addition, some investigators have reported that oil remobilization and spread may have affected their reference sites, which would additionally hinder the ability to detect impacts and track recovery (Turner et al. 2019). For all these reasons, we caution that our interpretations and predictions regarding vegetation recovery are conservative (i.e., may underestimate recovery time frames), and again, full recovery would not occur where marsh erosion was accelerated because of the spill, as oil‐induced erosion impacts would be permanent.

Marsh vegetation recovery from oil spills, particularly spills of medium crude oils in warm climates (such as the US Gulf Coast), may in many cases take only one to two years (Michel and Rutherford 2014), and as little as three to four years even when soil oiling and extensive plant mortality occur (Hester and Mendelssohn 2000, Mendelssohn et al. 2012). The US Gulf Coast oil spill literature prior to the *Deepwater Horizon* spill led DeLaune and Wright (2011) to observe and predict that “the long‐term effects of the [*Deepwater Horizon*] oil spill on marsh vegetation and the wetland ecosystem, as demonstrated by this review of the literature, are minimal and should wane through time due to the resiliency of these wetland ecosystems” (in fairness they offered various caveats as well). So why has full marsh vegetation recovery not occurred following the *Deepwater Horizon* spill? Factors leading to longer term or delayed marsh recovery from prior spills are summarized in Mendelssohn et al. (2012), Michel and Rutherford (2014), and Pezeshki and DeLaune (2015). Several of these factors probably contributed to plant death (both above and belowground) and delayed marsh vegetation recovery following the *Deepwater Horizon* spill, particularly for heavily oiled marshes. These factors include:

1. Thick (>1 cm) persistent oiling on the marsh substrate, including viscous emulsified oil, which weathers and biodegrades more slowly than liquid oil, and interferes with soil processes and gas exchange (Lin and Mendelssohn 2012, Michel et al. 2013, Zengel and Michel 2013, Zengel et al. 2015; see Nixon et al. 2016 who reported at least 73 km of heavier persistent oiling of marsh shorelines).
2. Complete or nearly complete oiling of the aboveground vegetation, which interferes with photosynthesis, gas exchange across plant tissues, and temperature regulation (Zengel et al. 2015, Lin et al. 2016, Hester et al. 2016; see Goovaerts et al. 2016 who estimated at least 109 km of marsh shorelines with >90% plant stem oiling in Louisiana).
3. Oiling during the peak vegetation growing season, from early June to September 2010 during the *Deepwater Horizon* spill, which impacts plants when their aboveground growth and metabolic processes are maximized and belowground reserves minimized, lessening their ability to generate new shoots (Mendelssohn et al. 2012, Zengel and Michel 2013, Zengel et al. 2015).
4. Plant species sensitivity to oiling, particularly relevant for marshes with a *Juncus roemerianus* component (Anderson and Hess 2012, Lin and Mendelssohn 2012, Lin et al. 2016, Willis et al. 2016).
5. Oiling of marsh subsurface soils, through oil burial, oil penetration of crab burrows and dead shoot/root channels, and mixing of oil into sediments during cleanup operations, further slowing oil weathering and biodegradation, interfering with soil processes, and exposing belowground plant tissues and the rhizosphere to oil (Lin and Mendelssohn 2012, Zengel and Michel 2013, Zengel et al. 2015, 2021, Levine et al. 2017).
6. Repetitive oiling, over at least several weeks until source control and on‐water spill response operations concluded in September 2010, resulting in re‐oiling of plants including laid over vegetation and new growth, and increased oil loading to the marsh surface and soils, further lessening the ability of plants to recover; localized remobilization of oil from the marsh substrate following storms also led to repetitive oiling (Lin and Mendelssohn 2012, Zengel and Michel 2013, Zengel et al. 2015).
7. Intensive or aggressive cleanup treatments with negative outcomes, such as physically damaging or removing the vegetation, mixing oil into the soils, and lowering the marsh surface; ~39% of heavily oiled sites and ~19% of all oiled sites in the current study were known to have had active cleanup treatments; however, positive marsh cleanup treatment outcomes were also observed during this spill, particularly when combined with planting (Zengel et al. 2015, 2021).

The primary factor shared among prior crude oil spills with marsh recovery time frames exceeding 10 years and the *Deepwater Horizon* spill was thick, persistent oiling on the marsh substrate (Michel and Rutherford 2014, Zengel et al. 2015). The *Amoco Cadiz* spill in addition had aggressive marsh cleanup treatments that were detrimental to marsh recovery, although planting also had beneficial effects in that case, partly offsetting cleanup impacts (Baca et al. 1987, Gilfillan et al. 1995, Mendelssohn et al. 2012, Michel and Rutherford 2014). Lastly, other stressors may have interacted with oiling exposure, further affecting the degree of impacts and vegetation recovery (DeLaune and Wright 2011, Mendelssohn et al. 2012, Silliman et al. 2012, Pezeshki and DeLaune 2015). Multiple stressors interacting with oiling and direct cleanup impacts may have included ongoing lack of sediment supply, marsh subsidence, relative sea level rise, high background rates of erosion, salinity changes during large freshwater releases enacted in reaction to the spill, several tropical storms and hurricanes, and drought conditions.

### 2 Recommendations for future studies

In addition to examining impacts and recovery, comparisons among vegetation metrics from our study are interesting from the perspective of which metrics were most valuable or meaningful, and therefore recommended for ongoing and future oil spill studies. Based on the data presented here, total aboveground biomass best integrated impacts and recovery across all the aboveground metrics and has logical ties to both marsh structure and function. However, examining species‐specific aboveground biomass is also vital because the lack of recovery in certain key species, or important species shifts (e.g., *Juncus roemerianus* to *Spartina alterniflora*), could be missed if only total aboveground biomass were examined. We also found belowground biomass to be a very important metric, particularly when considering full marsh recovery. Belowground biomass declined over time in contrast with recovery trends in nearly all the aboveground metrics and, therefore, was a strong indicator of ongoing impact and limited recovery. This is particularly insightful given the link between belowground biomass and marsh function and resilience, such as infauna and microbial communities, biogeochemistry, carbon sequestration, and soil shear strength and erodibility (Peterson et al. 2008, Macreadie et al. 2013, Lin et al. 2016, Silliman et al. 2016, Fleeger et al. 2019, 2020, Cagle et al. 2020). Belowground plant biomass is not often reported for oil spill studies, probably because of logistical and cost constraints as well as concerns about negatively impacting the marsh (see brief review in Silliman et al. 2016; Culbertson et al. 2008, Michel et al. 2009 are good examples of delayed belowground biomass recovery in coastal marshes from seven to 37 years following two refined fuel oil spills). We also note that belowground biomass was the least frequently measured metric among the *Deepwater Horizon* studies and datasets we analyzed, despite this being the key vegetation trait tied to erosion protection (Silliman et al. 2016). We encourage future oil spill studies to include belowground biomass measurements, when possible, particularly live belowground biomass, to provide deeper insights into full vegetation recovery, related functional attributes, and marsh resilience (in alignment with suggestions by Peterson et al. 2008). Coupling belowground biomass with standardized soil shear strength measurements is also recommended for both vegetation and erosion studies (as in Lin et al. 2016). As a further argument for biomass‐based metrics (aboveground and belowground), direct biomass measures are often advantageous for injury quantification and restoration scaling under NRDA (Baker et al. 2020). Given that belowground biomass development and maturity in restored marshes is also a long‐term process (Ebbets et al. 2019), more in‐depth and comparable information on belowground biomass recovery in oiled marshes would be valuable for NRDA injury determinations and restoration planning.

We also found the plant cover metrics to be valuable, particularly as this metric included the most available data, with all but one source in our study reporting cover values. Our plant cover metrics identified the degree of impacts and recovery trajectories comparably with other metrics, although in a few cases plant cover indicated slightly earlier recovery (mainly for total cover versus total aboveground biomass, see further discussion below). The ease and speed of collecting the plant cover metrics, and their non‐destructive nature, contribute to their widespread use, and these metrics are reasonable proxies for aboveground marsh biomass, structure, and function, particularly when paired with vegetation height, and especially when biomass cannot be collected due to logistical constraints (including the need to limit destructive sampling in some cases). A downside is that cover estimation can be subjective; however, approaches are available to address this, such as using cover estimation charts, quadrat quartering, gridded quadrats, standardized cover classes, multiple observers, point‐intercept methods, and digital photodocumentation and analysis.

As for total aboveground biomass, total percent plant cover is an important metric when multiple species are present, but it can also obscure species dynamics if not combined with species‐specific determinations. In our study, total plant cover indicated full recovery for the heavily oiled marsh edge at five years post‐spill, whereas *Spartina alterniflora* and *Juncus roemerianus* cover did not; both showed a lack of full recovery through five to six years post‐spill. This was due in part to shifts from the normally dominant vegetation, particularly *Spartina alterniflora*, to species such as *Paspalum vaginatum* and *Distichlis spicata*, with important implications for marsh structure, function, and resilience (Zengel et al. 2015, 2021). Similar species shifts from *Spartina alterniflora* to *Distichlis spicata* in heavily oiled areas were also observed by others (Beland et al. 2016, Johnson et al. 2018, Cagle et al. 2020), although this topic was not often examined or reported in most *Deepwater Horizon* marsh studies. Willis et al. (2016) indicated possible species shifts from *Juncus roemerianus* to a mix of other species including *Distichlis spicata*, *Spartina patens*, and *Salicornia* sp., although this subject was not addressed in detail. We encourage future examination of plant species composition changes and interactions following oil spills (in alignment with Peterson et al. 2008, as well as Pezeshki and DeLaune 2015, particularly for mixed species marshes; see also Hughes et al. 2018 and Zerebecki et al. 2021 for mixed marsh and mangrove habitats).

We advise caution when using stem density alone to define salt marsh impacts and recovery, because our study indicated that this metric can be misleading if reported or interpreted in the absence of other metrics (a finding somewhat in contrast with Peterson et al. 2008). In our study integrating multiple datasets, stem density, particularly *Spartina alterniflora* stem density, did not fully capture either the degree of impacts or longer recovery time frames indicated by the other plant metrics, although we did note that increased stem density coupled with ongoing impacts to other metrics, such as aboveground biomass, may point to reductions in plant health. For aboveground metrics, we recommend combining stem density metrics (when used) with plant cover, aboveground biomass, and vegetation height measurements.

We suggest that future studies use fixed sampling locations supplemented with additional sampling sites if shoreline position changes over the duration of the study. The positions of all sampling locations should be recorded and tracked through time as accurately as possible (and reported as fixed or non‐fixed, as applicable). In addition to horizontal position, marsh surface elevation should be recorded for sampling locations, when possible, as elevation can influence a variety of vegetation metrics including species composition, particularly in relation to shoreline retreat. Shoreline erosion measurements should be closely integrated into oiled vegetation sampling, and vice versa, and used to interpret findings. Both field‐based and remotely sensed erosion measurements can be valuable, including when used in combination to look at longer time periods, such as pre‐spill background rates versus post‐spill changes and shifting baselines.

Initial and subsequent oiling conditions need to be carefully documented for study sites during each sampling event, addressing oil both on the vegetation and on or in the marsh soils, including site‐specific measurements or descriptions of oiling width across shore, oiling height on the vegetation, horizontal and vertical oiling percent cover, oiling thickness, oiling characteristics (liquid oil, emulsified oil, weathered oil mixed with sediment, etc.), and observations of subsurface oiling (i.e., oil that has penetrated the soils or become buried) (for methods and definitions see Michel et al. 2013 and Nixon et al. 2016, including references and supplementary information therein). Oil chemistry sampling coupled with the above and closely tied to sampling locations is also valuable if sampled with sufficient intensity. Oil chemistry metrics of greatest interest include polycyclic aromatic hydrocarbons to assess oil toxicity and track weathering of the oil over time, and biomarkers if there is a concern about the source of the oil. Background oiling levels also need to be considered, particularly in areas with chronic sources such as frequent small spills and heavy industrial activity. Oil chemistry data should not be used to the exclusion of the oiling condition metrics described above, as this would provide an incomplete picture of exposure pathways and impacts, especially in the early periods after a spill. For studies that begin later, after oiling conditions have changed, researching the initial oiling characteristics to the greatest degree possible, and examining prior shoreline erosion using aerial photographs, would be helpful in interpreting impacts and recovery.

### 3 Summary and conclusions

In conclusion, we found that the *Deepwater Horizon* oil spill had substantial multiyear impacts on salt marsh vegetation, with full recovery exceeding seven years, and likely to extend to 10 years or more, particularly in heavily oiled marshes, with impacts being permanent in cases in which oiling has led to increased marsh erosion. Our findings contribute new and expanded knowledge on salt marsh structure, function, and resilience following the *Deepwater Horizon* oil spill, informing future environmental impact analysis, contingency planning, emergency response, damage assessment, and restoration efforts, particularly impact and recovery trajectories and restoration scaling related to oil spills. *Spartina alterniflora* and *Juncus roemerianus* serve as the defining foundation species for salt marshes in the region, providing physical structure, primary productivity, habitat cover, organic matter and detritus, food web support, nutrient cycling, and development and stabilization of marsh soils. Accordingly, the observed vegetation impacts and delayed recovery are likely to have exerted substantial influence on numerous ecosystem processes and associated species. Marsh oiling impacts were most severe and long‐lasting at the marsh edge, which is particularly relevant for ecosystem functions such as shoreline stabilization, coastal protection, and marsh‐dependent fisheries support. Although all or nearly all existing salt marsh vegetation studies examining the *Deepwater Horizon* oil spill have ceased or are winding down, we encourage long‐term vegetation monitoring studies to continue, when applicable, so that marsh recovery can be tracked to conclusion. For both ongoing monitoring and future spills, we recommend that marsh vegetation studies incorporate multiple metrics, including live belowground biomass, to provide the fullest picture of marsh impacts, functionality, recovery, and resilience. Coupling vegetation sampling with site‐specific oiling data and erosion measurements from the onset of oiling through recovery is also recommended.

## Supporting information

Appendix S1

Appendix S2

## Data Availability

Data (Zengel et al. 2020) are publicly available through the Gulf of Mexico Research Initiative Information & Data Cooperative (GRIIDC) at https://doi.org/10.7266/n7‐cn03‐z792
