# Supplementary material for: Meta‐analysis of salt marsh vegetation impacts and recovery: a synthesis following the Deepwater Horizon oil spill
Source: Ecol Appl. 2021 Dec 8;32(1):e02489. doi: 10.1002/eap.2489 (PMC9285535; doi:10.1002/eap.2489)
Supplement: Supplementary file 1 — Appendix S1 [file EAP-32-0-s001.pdf]

Supporting Information. Zengel et al. 2021. Meta-analysis of salt marsh vegetation impacts and recovery: a synthesis following the *Deepwater Horizon* oil spill. Ecological Applications.

Appendix S1: Supporting tables and figures

Appendix S1: Table S1. Summary of marsh vegetation data sources and metrics used in the meta-analyses. Dominant species are *Spartina alterniflora* and *Juncus roemerianus*.

**Source**	**Years**	**Marsh Zones**	**Dominant Species**	**Plant Cover**	**Stem Density**	**Vegetation Height**	**Aboveground Biomass**	**Belowground Biomass**	**Primary References**
Hester	2010-2013	Edge, Interior	*Spartina*	X	X	X	X	X	Hester et al. 2016, and unpublished data
Silliman	2010-2013	Edge, Interior	*Spartina*	X					Silliman et al. 2012, and unpublished data
McClenachan	2010-2012	Edge	*Spartina*	X					McClenachan et al. 2013
Willis	2011-2013	Edge, Interior	*Juncus, Spartina*,	X	X	X	X	X	Willis et al. 2016, and unpublished data
Zengel (A)	2011	Edge, Interior	*Spartina*, *Juncus*	X		X			Zengel et al. 2016a, and unpublished data
Lin	2011-2017	Edge	*Spartina*, *Juncus*		X		X	X*	Lin and Mendelssohn 2012, Lin et al. 2016, and unpublished data
Zengel (B)	2011-2016	Edge	*Spartina*, *Juncus*	X		X			Zengel et al. 2015, 2021, and unpublished data
Rabalais	2013	Edge, Interior	*Spartina*	X		X	X		Rabalais 2016
Hughes	2015-2016	Edge	*Spartina*	X	X	X			Hughes 2016, Zerebecki et al. 2021
Cebrian	2015-2016	Edge	*Spartina*	X	X				Cebrian and Goff 2017

*Belowground biomass data for 2011-2014, subsequent data withheld for pending manuscript.

Appendix S1: Table S2. Summary of maximum mean impact degree*, In[RR] (% reduction), by vegetation metric, oiling category, and marsh zone. Species are *Spartina alterniflora* and *Juncus roemerianus*. For biomass, AG is aboveground and BG is belowground.

Vegetation Metric	All Oiled Sites Marsh Edge	All Oiled Sites Marsh Interior	Heavily Oiled Sites Marsh Edge	Heavily Oiled Sites Marsh Interior
Total plant cover	-1.37 (75%)	-0.36 (30%)	-2.11 (88%)	-0.36 (30%)
Spartina plant cover	-1.40 (75%)	-0.40 (33%)	-2.11 (88%)	-0.33 (28%)
Juncus plant cover	-7.44 (100%)	-2.10 (88%)	-7.45 (100%)	single observation
Total stem density	-0.43 (35%)	not impacted	-1.01 (63%)	not impacted
Spartina stem density	-0.41 (34%)	not impacted	-0.56 (43%)	-0.64 (47%)
Juncus stem density	-1.94 (86%)	-2.31 (90%)	-10.93 (100%)	no data
Vegetation height	-0.49 (39%)	-0.43 (35%)	-0.65 (48%)	not impacted
Total AG biomass	-0.65 (48%)	-0.48 (38%)	-1.18 (69%)	-0.52 (41%)
Spartina AG biomass	-0.58 (44%)	-0.69 (50%)	-1.07 (66%)	-1.07 (66%)
Juncus AG biomass	-1.92 (85%)	-2.75 (94%)	-11.18 (100%)	no data
BG biomass	-0.66 (48%)	-0.38 (32%)	-1.17 (69%)	-0.23 (20%)

*The greatest degree of impact observed in any one year following the spill, averaged across sources, typically corresponds to initial or early impacts, but sometimes includes delayed or worsening impacts in the absence of recovery.

Appendix S1: Table S3. Random effects model comparisons of oiling and reference conditions by vegetation metric, oiling category, and marsh zone across all years combined. Species are *Spartina alterniflora* and *Juncus roemerianus*. B-H refers to adjusted p-values for multiple comparisons based on the Benjamini-Hochberg method.

**Vegetation Metric**	**Oiling Category**	**Marsh Zone**	**Mean effect (lnRR) and 90% CI**	**t**	**df**	**se**	**p**	**B-H adj. p**
Total plant cover	All Oiled Sites	Edge	-0.43 (-0.66, -0.20)	-3.21	24	0.13	0.0037	0.0101
		Interior	-0.21 (-0.34, -0.09)	-3.08	11	0.07	0.0104	0.0225
	Heavily Oiled Sites	Edge	-0.84 (-1.20, -0.49)	-4.25	13	0.20	0.0009	0.0048
		Interior	-0.18 (-0.36, 0.00)	-1.86	7	0.10	0.1059	0.1240
Spartina plant cover	All Oiled Sites	Edge	-0.64 (-1.00, -0.27)	-2.98	23	0.21	0.0066	0.0160
		Interior	-0.26 (-0.50, -0.02)	-2.00	10	0.13	0.0736	0.0984
	Heavily Oiled Sites	Edge	-1.13 (-1.70, -0.56)	-3.49	13	0.32	0.0040	0.0102
		Interior	-0.27 (-0.49, -0.06)	-2.43	7	0.11	0.0455	0.0778
Juncus plant cover	All Oiled Sites	Edge	-5.17 (-6.68, -3.65)	-6.33	8	0.82	0.0002	0.0023
		Interior	-1.53 (-2.98, -0.08)	-2.48	3	0.62	0.0894	0.1145
	Heavily Oiled Sites	Edge	-5.99 (-7.48, -4.50)	-8.11	5	0.74	0.0005	0.0032
		Interior	-0.03 (single obs.)	--	--	--	--	--
Total stem density	All Oiled Sites	Edge	-0.09 (-0.28, 0.10)	-0.81	17	0.11	0.4298	0.4637
		Interior	-0.01 (-0.16, 0.15)	-0.08	6	0.08	0.9412	0.9412
	Heavily Oiled Sites	Edge	-0.82 (-1.11, -0.53)	-5.12	10	0.16	0.0005	0.0032
		Interior	-0.09 (-0.31, 0.13)	-0.94	3	0.09	0.4176	0.4628
Spartina stem density	All Oiled Sites	Edge	0.27 (0.07, 0.48)	2.32	17	0.12	0.0330	0.0588
		Interior	0.04 (-0.25, 0.33)	0.29	6	0.15	0.7832	0.8028
	Heavily Oiled Sites	Edge	0.05 (-0.22, 0.32)	0.32	10	0.15	0.7567	0.7955
		Interior	-0.28 (-0.56, 0.00)	-2.32	3	0.12	0.1028	0.1239
Juncus stem density	All Oiled Sites	Edge	-0.95 (-1.4, -0.50)	-3.89	9	0.24	0.0037	0.0101
		Interior	-2.03 (-3.68, -0.38)	-3.59	2	0.57	0.0696	0.0984
	Heavily Oiled Sites	Edge	-9.81 (-11.26, -8.36)	-13.13	6	0.75	0.0000	0.0002
		Interior	no data	--	--	--	--	--
Vegetation height	All Oiled Sites	Edge	-0.28 (-0.41, -0.15)	-3.81	14	0.07	0.0019	0.0079
		Interior	-0.27 (-0.50, -0.03)	-2.08	8	0.13	0.0712	0.0984
	Heavily Oiled Sites	Edge	-0.33 (-0.50, -0.15)	-3.44	8	0.09	0.0089	0.0202
		Interior	-0.08 (-0.22, 0.05)	-1.33	4	0.06	0.2541	0.2894
Total aboveground biomass	All Oiled Sites	Edge	-0.38 (-0.53, -0.23)	-4.38	14	0.09	0.0006	0.0037
		Interior	-0.33 (-0.47, -0.19)	-4.36	7	0.08	0.0033	0.0101
	Heavily Oiled Sites	Edge	-0.84 (-1.10, -0.58)	-5.87	10	0.14	0.0002	0.0021
		Interior	-0.32 (-0.58, -0.05)	-2.82	3	0.11	0.0668	0.0984

Spartina aboveground biomass	All Oiled Sites	Edge	-0.15 (-0.31, 0.00)	-1.75	14	0.09	0.1018	0.1239
		Interior	-0.38 (-0.65, -0.12)	-2.74	7	0.14	0.0290	0.0540
	Heavily Oiled Sites	Edge	-0.52 (-0.75, -0.29)	-4.06	10	0.13	0.0023	0.0085
		Interior	-0.67 (-1.18, -0.16)	-3.08	3	0.22	0.0540	0.0885
Juncus aboveground biomass	All Oiled Sites	Edge	-0.95 (-1.4, -0.50)	-3.89	9	0.24	0.0037	0.0101
		Interior	-2.03 (-3.68, -0.38)	-3.59	2	0.57	0.0696	0.0984
	Heavily Oiled Sites	Edge	-9.83 (-11.45, -8.21)	-14.84	6	0.66	0.000	0.0002
		Interior	no data	--	--	--	--	--
Belowground biomass	All Oiled Sites	Edge	-0.47 (-0.67, -0.27)	-4.29	10	0.11	0.0016	0.0072
		Interior	-0.24 (-0.39, -0.10)	-3.21	6	0.08	0.0184	0.0359
	Heavily Oiled Sites	Edge	-0.88 (-1.37, -0.39)	-3.42	7	0.26	0.0111	0.0227
		Interior	-0.15 (-0.29, -0.02)	-2.69	3	0.06	0.0744	0.0984

Appendix S1: Table S4. Summary of impact duration/recovery time frames (years) by vegetation metric, oiling category, and marsh zone. Species are *Spartina alterniflora* and *Juncus roemerianus*. For biomass, AG is aboveground and BG is belowground.

**Vegetation Metric**	**All Oiled Sites Marsh Edge**	**All Oiled Sites Marsh Interior**	**Heavily Oiled Sites Marsh Edge**	**Heavily Oiled Sites Marsh Interior**
Total plant cover	5 years	3 years	5 years	3 years
Spartina plant cover	6 years	3 years	not recovered (>6 years)	3 years
Juncus plant cover	not recovered (>5 years)	not recovered (>3 years)	not recovered (>5 years)	single observation
Total stem density	3 years	not impacted	not recovered (>7 years)	not impacted
Spartina stem density	1 year	not impacted	3 years	not recovered (>3 years)
Juncus stem density	not recovered (>7 years)	not recovered (>3 years)	not recovered (>7 years)	no data
Vegetation height	not recovered (>6 years)	not recovered (>3 years)	not recovered (>6 years)	not impacted
Total AG biomass	not recovered (>7 years)	not recovered (>3 years)	not recovered (>7 years)	3 years
Spartina AG biomass	4 years	not recovered (>3 years)	4 years	not recovered (>3 years)
Juncus AG biomass	not recovered (>7 years)	not recovered (>3 years)	not recovered (>7 years)	no data
BG biomass	not recovered (>4 years)	not recovered (>3 years)	not recovered (>4 years)	not recovered (>3 years)

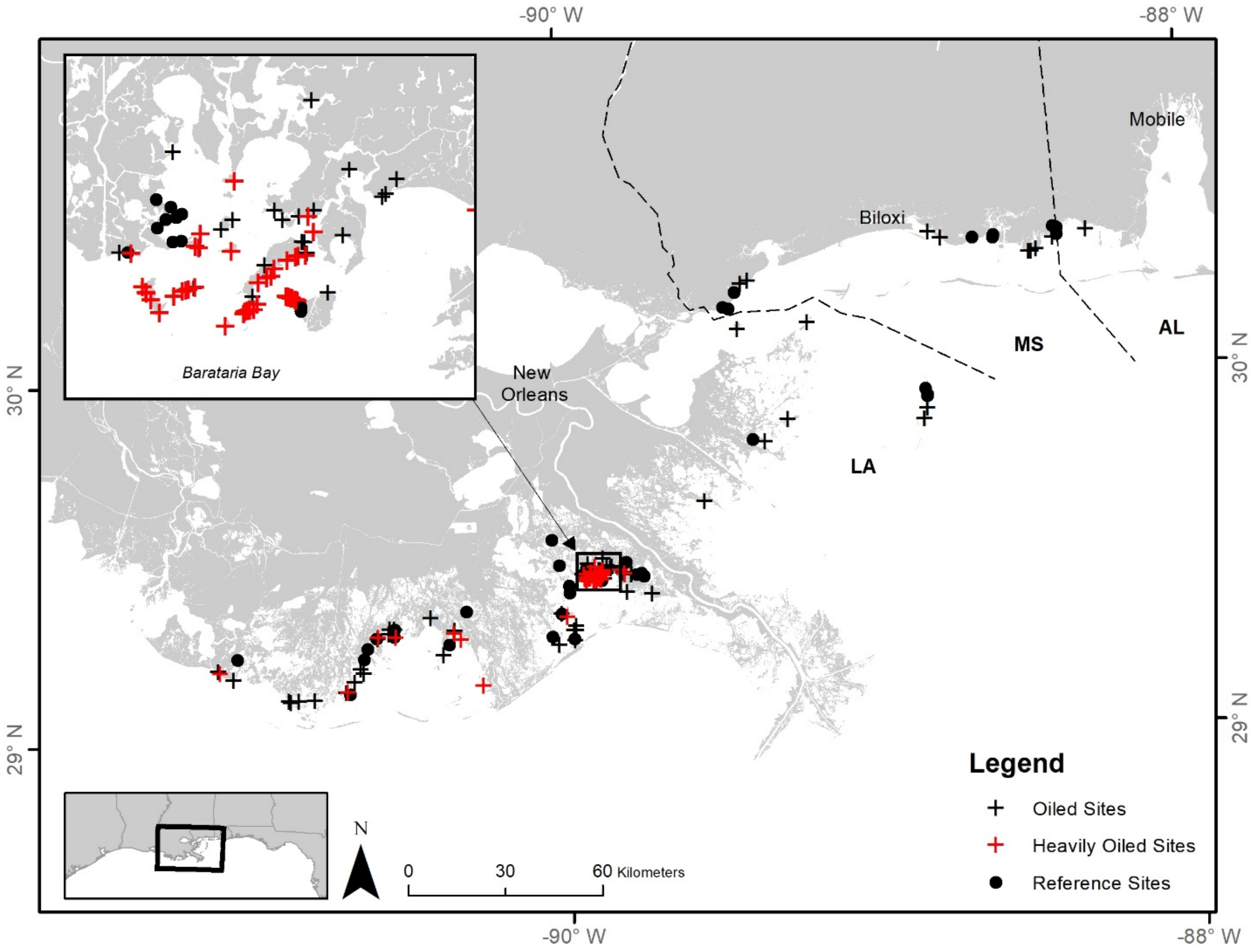

Appendix S1: Figure S1. Study area map showing reference, oiled, and heavily oiled sampling sites from all data sources contributing to the meta-analyses.

Literature Cited

Cebrian J, Goff J. 2017. Site characterization using estimates of vegetative cover, and assessment of marsh/mangrove above and below ground productivity, Chandeleur Islands 2015-2016. GoMRI GRIIDC datasets, doi:10.7266/N7JS9NH9, doi:10.7266/N7930RJH, and doi:10.7266/N7DR2SZJ.

Hester MW, Willis JM, Rouhani S, Steinhoff MA, Baker MC. 2016. Impacts of the *Deepwater Horizon* oil spill on the salt marsh vegetation of Louisiana. Environmental Pollution 216:361-370.

Hughes R. 2016. Impact of DWH oil spill on *Spartina alterniflora* in the Chandeleur Islands, Louisiana, June 2015- September 2016. GoMRI GRIIDC dataset, doi:10.7266/N7QJ7FBZ.

Lin Q, Mendelssohn IA. 2012. Impacts and recovery of the *Deepwater Horizon* oil spill on vegetation structure and function of coastal salt marshes in the northern Gulf of Mexico. Environmental Science & Technology 46:3737−3743.

Lin Q, Mendelssohn IA, Graham SA, Hou A, Fleeger JW, Deis DR. 2016. Response of salt marshes to oiling from the *Deepwater Horizon* spill: implications for plant growth, soil surface-erosion, and shoreline stability. Science of the Total Environment 557:369-377.

McClenachan G, Turner RE, Tweel AW. 2013. Effects of oil on the rate and trajectory of Louisiana marsh shoreline erosion. Environmental Research Letters 8(4):044030.

Rabalais N. 2016. Above ground plant biomass, canopy height and estimated percent cover supporting marsh and subtidal benthic community and marsh invertebrate distribution studies in paired oiled/unoiled sites in coastal Louisiana in spring and fall 2013. GoMRI GRIIDC dataset, doi:10.7266/N7X34VD2.

Silliman BR, van de Koppel J, McCoy MW, Kasozi GN, Earl K, Adams PN, Zimmerman AR. 2012. Degradation and resilience in Louisiana salt marshes after the BP–*Deepwater Horizon* oil spill. Proceedings of the National Academy of Sciences USA 109:11234-11239.

Willis JM, Hester MW, Rouhani S, Steinhoff MA, Baker MC. 2016. Field assessment of the impacts of *Deepwater Horizon* oiling on coastal marsh vegetation of Mississippi and Alabama. Environmental Toxicology and Chemistry 35(11):2791-2797.

Zengel S, Bernik BM, Rutherford N, Nixon Z, Michel J. 2015. Heavily oiled salt marsh following the *Deepwater Horizon* oil spill, ecological comparisons of shoreline cleanup treatments and recovery. PLOS One 10:e0132324.

Zengel S, Montague CL, Pennings SC, Powers SP, Steinhoff M, Fricano G, Schlemme C, Zhang M, Oehrig J, Nixon Z, Rouhani S, Michel J. 2016a. Impacts of the *Deepwater Horizon* oil spill on salt marsh periwinkles (*Littoraria irrorata*). Environmental Science & Technology 50:643−652.

Zengel S, Rutherford N, Bernik BM, Weaver J, Zhang M, Nixon Z, Michel J. 2021. Planting after shoreline cleanup treatment improves salt marsh vegetation recovery following the *Deepwater Horizon* oil spill. Ecological Engineering 169:106288.

Zerebecki RA, Hughes AR, Goff J, Hanley TC, Scheffel W, Heck K. 2021. Effect of foundation species composition and oil exposure on wetland communities across multiple trophic levels. Marine Ecology Progress Series 662:53-68.
