# Supplementary material for: Meta‐analysis of salt marsh vegetation impacts and recovery: a synthesis following the Deepwater Horizon oil spill
Source: Ecol Appl. 2021 Dec 8;32(1):e02489. doi: 10.1002/eap.2489 (PMC9285535; doi:10.1002/eap.2489)
Supplement: Supplementary file 2 — Appendix S2 [file EAP-32-0-s002.pdf]

Supporting Information. Zengel et al. 2021. Meta-analysis of salt marsh vegetation impacts and recovery: a synthesis following the *Deepwater Horizon* oil spill. Ecological Applications.

Appendix S2: Meta-analysis for lightly and moderately oiled sites

Introduction

Analyses for lightly oiled and moderately oiled sites are provided below, similar in format to the analyses for the "all oiled" sites and heavily oiled sites presented in the main body of the paper. Studies with groups of sites reliably classified as lightly oiled (one study) and moderately oiled (three studies) were limited in number and duration of data. These analyses are provided with limited interpretation due to small sample size. These data are also included in the "all oiled" sites analyses in the main body of the paper (used in combination with other data). If comparing figures across oiling levels in the main body of the paper with the supplemental figures, be aware of differences in scaling of the x and y axes in some instances.

Plant Cover

Impacts to plant percent cover were qualitatively observed for the moderately oiled sites, with degrees of impact and recovery patterns somewhat similar to the all oiled sites, to a lesser degree in some instances, but including the absence of full recovery through the duration of data in most cases, two to three years post-spill (Appendix S2: Figure S1, Table S1). Plant cover was similarly impacted in the lightly oiled sites in the marsh interior, but not at the marsh edge (Appendix S2: Figure S1, Table S1).

Stem Density

Impacts to stem density were qualitatively observed in some cases for the moderately oiled sites, with degrees of impact and patterns of recovery similar to the all oiled sites, although *Juncus roemerianus* stem density at the marsh edge recovered more quickly (Appendix S2: Figure S2, Table S1). In the lightly oiled sites, *Spartina alterniflora* stem density indicated a possible qualitative impact and recovery trend in the marsh interior, but stem density was otherwise not impacted (Appendix S2: Figure S2, Table S1).

Vegetation Height

Impacts on vegetation height were qualitatively observed for the moderately oiled sites, with degrees of impact and patterns of recovery similar to the all oiled sites, including the absence of full recovery through the duration of our data, three years post-spill (Appendix S2: Figure S3, Table S1). Vegetation height did not appear to be impacted in the lightly oiled sites (Appendix S2: Figure S3, Table S1).

Aboveground Biomass

Impacts to aboveground biomass were qualitatively observed for the moderately oiled sites, with degrees of impact and patterns of recovery similar to the all oiled sites, but with recovery occurring more quickly in some cases (Appendix S2: Figure S4, Table S1). Aboveground biomass was similarly impacted in the lightly oiled sites in the marsh interior, but not at the marsh edge (Appendix S2: Figure S4, Table S1).

Belowground Biomass

Impacts to belowground biomass were quantitatively observed at the marsh edge for the moderately oiled sites ($p = 0.0026$; B-H adjusted $p = 0.0980$), and qualitatively observed for the marsh interior, with degrees of impact similar to the all oiled sites, including the absence of full recovery through the duration of our data, four years post-spill (Appendix S2: Figure S5, Table S1). Belowground biomass was not impacted in the lightly oiled sites (Appendix S2: Figure S5, Table S1).

Summary

Although the data were limited and statistical comparisons less compelling in most instances, impacts and recovery in the moderately oiled sites were often similar to the all oiled sites, although in a few cases the degree of impacts were lesser and recovery quicker in the

moderately oiled sites. Regardless, full vegetation recovery in the moderately oiled sites was not complete over the duration of our data including, importantly, for belowground biomass at the marsh edge. Impacts to the lightly oiled sites were also observed, but to a lesser degree and across fewer metrics, as might be expected. In contrast to sites with higher oiling levels, impacts to lightly oiled sites appeared to be more common in the marsh interior, possibly due to higher oiling levels for some sites in the marsh interior (see Hester et al. 2016, their Table 2, 0-10% stem oiling, zone 1 versus 2). Perhaps for lighter oiling, natural flushing by tides and waves removed more oil from the marsh edge than the marsh interior, or otherwise promoted faster recovery at the marsh edge.

Literature Cited

Hester MW, Willis JM, Rouhani S, Steinhoff MA, Baker MC. 2016. Impacts of the *Deepwater Horizon* oil spill on the salt marsh vegetation of Louisiana. Environmental Pollution 216:361-370.

Appendix S2: Table S1. Random or fixed effects model comparisons of oiling and reference conditions by vegetation metric, oiling category, and marsh zone across all years combined for lightly and moderately oiled sites. A fixed effects model was used where only one data source was contributing to a comparison (for the lightly oiled sites). Species are *Spartina alterniflora* and *Juncus roemerianus*. B-H refers to adjusted p-values for multiple comparisons based on the Benjamini-Hochberg method.

Vegetation Metric	Oiling Category	Marsh Zone	Mean effect (lnRR) and 90% CI	t	df	se	p value	B-H adj. p
Total Plant Cover	Lightly Oiled Sites	Edge	-0.09 (-0.40, 0.22)	-0.66	3	0.13	0.5574	0.6833
		Interior	-0.40 (-0.67, -0.13)	-3.44	3	0.11	0.0412	0.2235
	Moderately Oiled Sites	Edge	-0.67 (-1.03, -0.31)	-3.59	6	0.19	0.0115	0.1458
		Interior	-0.18 (-0.31, -0.05)	-2.78	6	0.07	0.0319	0.2098
Spartina Plant Cover	Lightly Oiled Sites	Edge	-0.22 (-0.60, 0.16)	-1.36	3	0.16	0.2681	0.3919
		Interior	-0.49 (-0.80, -0.18)	-3.75	3	0.13	0.0331	0.2098
	Moderately Oiled Sites	Edge	-1.45 (-3.35, 0.46)	-1.48	6	0.98	0.1902	0.3475
		Interior	-0.66 (-1.01, -0.30)	-3.60	6	0.18	0.0114	0.1458
Juncus Plant Cover	Lightly Oiled Sites	Edge	no data	--	--	--	--	--
		Interior	no data	--	--	--	--	--
	Moderately Oiled Sites	Edge	-3.35 (-9.03, 2.33)	-1.72	2	1.95	0.2273	0.3756
		Interior	-1.66 (-3.38, 0.05)	-2.84	2	0.59	0.1049	0.2491
Total Stem Density	Lightly Oiled Sites	Edge	0.07 (-0.41, 0.55)	0.34	3	0.20	0.7575	0.8106
		Interior	-0.12 (-0.49, 0.25)	-0.77	3	0.16	0.4998	0.6331
	Moderately Oiled Sites	Edge	-0.09 (-0.27, 0.08)	-0.92	13	0.10	0.3745	0.5082
		Interior	0.11 (-0.04, 0.27)	1.45	6	0.08	0.1965	0.3475
Spartina Stem Density	Lightly Oiled Sites	Edge	-0.25 (-0.61, 0.11)	-1.63	3	0.15	0.2021	0.3475
		Interior	-0.19 (-0.51, 0.12)	-1.42	3	0.13	0.2494	0.3791
	Moderately Oiled Sites	Edge	0.16 (-0.04, 0.37)	1.40	13	0.12	0.1854	0.3475
		Interior	-0.07 (-0.47, 0.33)	-0.35	6	0.21	0.7368	0.8106
Juncus Stem Density	Lightly Oiled Sites	Edge	no data	--	--	--	--	--
		Interior	no data	--	--	--	--	--
	Moderately Oiled Sites	Edge	-0.38 (-0.81, 0.06)	-1.60	9	0.24	0.1447	0.3055
		Interior	-1.64 (-3.32, 0.03)	-2.86	2	0.57	0.1036	0.2491
Vegetation height	Lightly Oiled Sites	Edge	0.04 (-0.14, 0.22)	0.49	3	0.08	0.6568	0.7800
		Interior	0.03 (-0.17, 0.23)	0.36	3	0.09	0.7452	0.8106
	Moderately Oiled Sites	Edge	-0.12 (-0.31, 0.06)	-1.32	5	0.09	0.2442	0.3791
		Interior	-0.33 (-0.63, -0.02)	-2.08	6	0.16	0.0823	0.2406

Total aboveground biomass	Lightly Oiled Sites	Edge	-0.04 (-0.53, 0.44)	-0.20	3	0.21	0.8519	0.8749
		Interior	-0.31 (-0.57, -0.04)	-2.69	3	0.11	0.0743	0.2406
	Moderately Oiled Sites	Edge	-0.23 (-0.41, -0.04)	-2.13	13	0.11	0.0530	0.2239
		Interior	-0.24 (-0.39, -0.10)	-3.30	6	0.07	0.0164	0.1559
Spartina aboveground biomass	Lightly Oiled Sites	Edge	-0.24 (-0.69, 0.20)	-1.28	3	0.19	0.2906	0.4090
		Interior	-0.42 (-0.73, -0.12)	-3.24	3	0.13	0.0477	0.2239
	Moderately Oiled Sites	Edge	-0.03 (-0.23, 0.16)	-0.30	13	0.11	0.7679	0.8106
		Interior	-0.38 (-0.78, 0.02)	-1.84	6	0.21	0.1159	0.2592
Juncus aboveground biomass	Lightly Oiled Sites	Edge	no data	--	--	--	--	--
		Interior	no data	--	--	--	--	--
	Moderately Oiled Sites	Edge	-0.47 (-0.90, -0.04)	-2.00	9	0.24	0.0762	0.2406
		Interior	-1.72 (-3.44, -0.00)	-2.93	2	0.59	0.0996	0.2491
Belowground biomass	Lightly Oiled Sites	Edge	-0.08 (-0.29, 0.14)	-0.82	3	0.09	0.4730	0.6199
		Interior	0.01 (-0.18, 0.20)	0.11	3	0.08	0.9192	0.9192
	Moderately Oiled Sites	Edge	-0.34 (-0.50, -0.19)	-3.99	10	0.09	0.0026	0.0980
		Interior	-0.18 (-0.35, -0.02)	-2.14	6	0.08	0.0762	0.2406

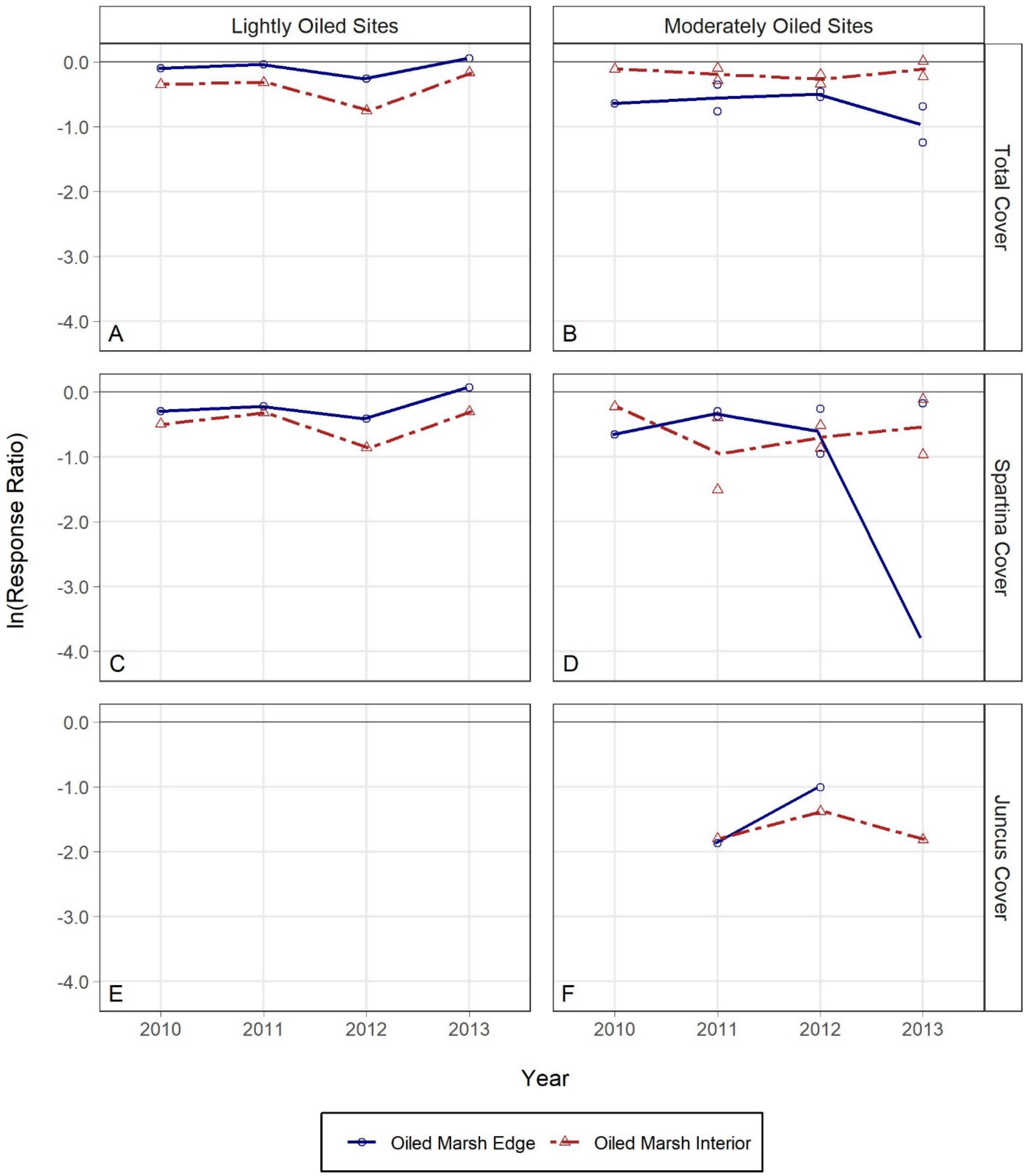

Appendix S2: Figure S1. Response ratios (ln[RR]) (oiled/reference) for plant cover (%) by marsh zone and year for: (A) total cover for lightly oiled sites, (B) total cover for moderately oiled sites, (C) *Spartina alterniflora* cover for lightly oiled sites, (D) *Spartina alterniflora* cover

for moderately oiled sites, (E) *Juncus roemerianus* cover for lightly oiled sites (no data), and (F) *Juncus roemerianus* cover for moderately oiled sites. Lines connect mean annual ln(RR) values by marsh zone across years. ln(RR) values from each contributing source are plotted as open symbols. ln(RR) values less than zero indicate reductions in metrics for oiled sites compared to reference (= impacts). As a guide to interpreting degree of effects, ln(RR) = -0.7 is a ~50% reduction for oiled sites relative to reference, ln(RR) = -2.3 is a ~90% reduction, and ln(RR) = -4.6 is a ~99% reduction.

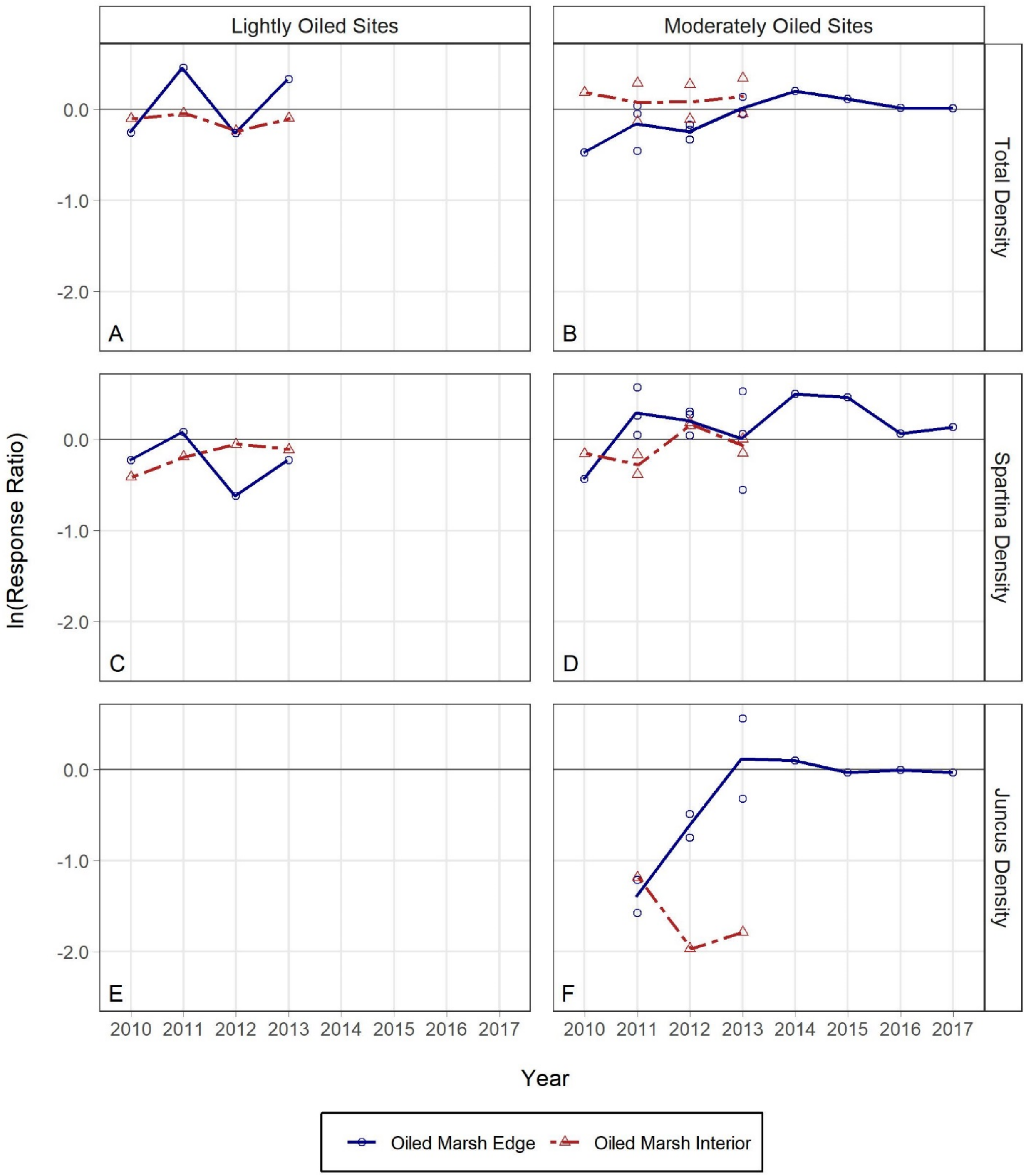

Appendix S2: Figure S2. Response ratios (ln[RR]) (oiled/reference) for stem density (no. m^{-2}) by marsh zone and year for: (A) total density for lightly oiled sites, (B) total density for moderately oiled sites, (C) *Spartina alterniflora* density for lightly oiled sites, (D) *Spartina*

alterniflora density for moderately oiled sites, (E) *Juncus roemerianus* density for lightly oiled sites (no data), and (F) *Juncus roemerianus* density for moderately oiled sites. Lines connect mean annual ln(RR) values by marsh zone across years. ln(RR) values from each contributing source are plotted as open symbols. ln(RR) values less than zero indicate reductions in metrics for oiled sites compared to reference (= impacts). As a guide to interpreting degree of effects, ln(RR) = -0.7 is a ~50% reduction for oiled sites relative to reference, ln(RR) = -2.3 is a ~90% reduction, and ln(RR) = -4.6 is a ~99% reduction.

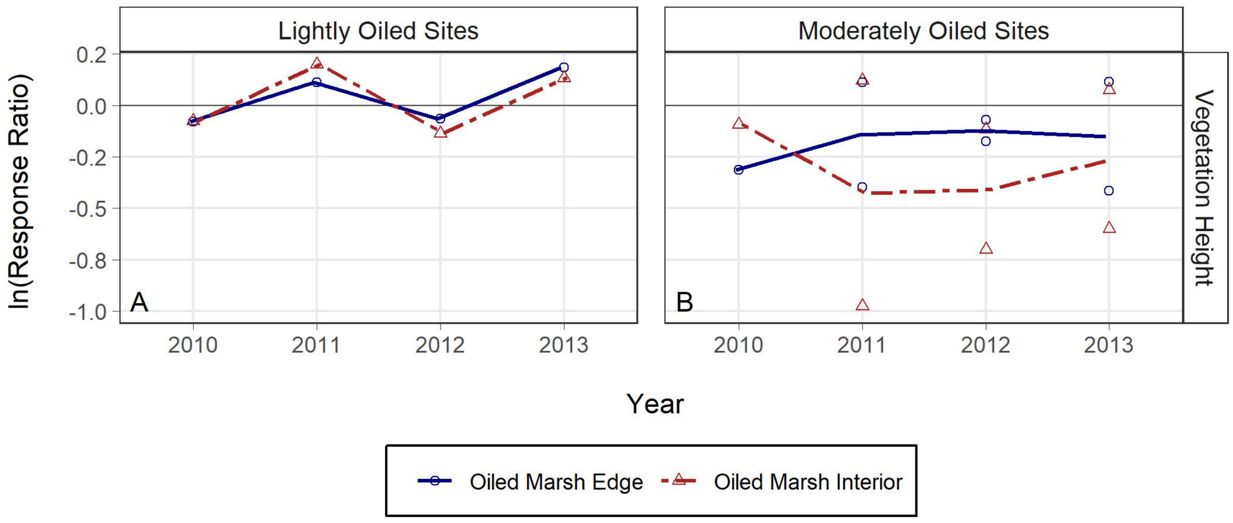

Appendix S2: Figure S3. Response ratios (ln[RR]) (oiled/reference) for vegetation height (cm) by marsh zone and year for: (A) lightly oiled sites, (B) moderately oiled sites. Lines connect mean annual ln(RR) values by marsh zone across years. ln(RR) values from each contributing source are plotted as open symbols. ln(RR) values less than zero indicate reductions in metrics for oiled sites compared to reference (= impacts). As a guide to interpreting degree of effects, ln(RR) = -0.7 is a ~50% reduction for oiled sites relative to reference, ln(RR) = -2.3 is a ~90% reduction, and ln(RR) = -4.6 is a ~99% reduction.

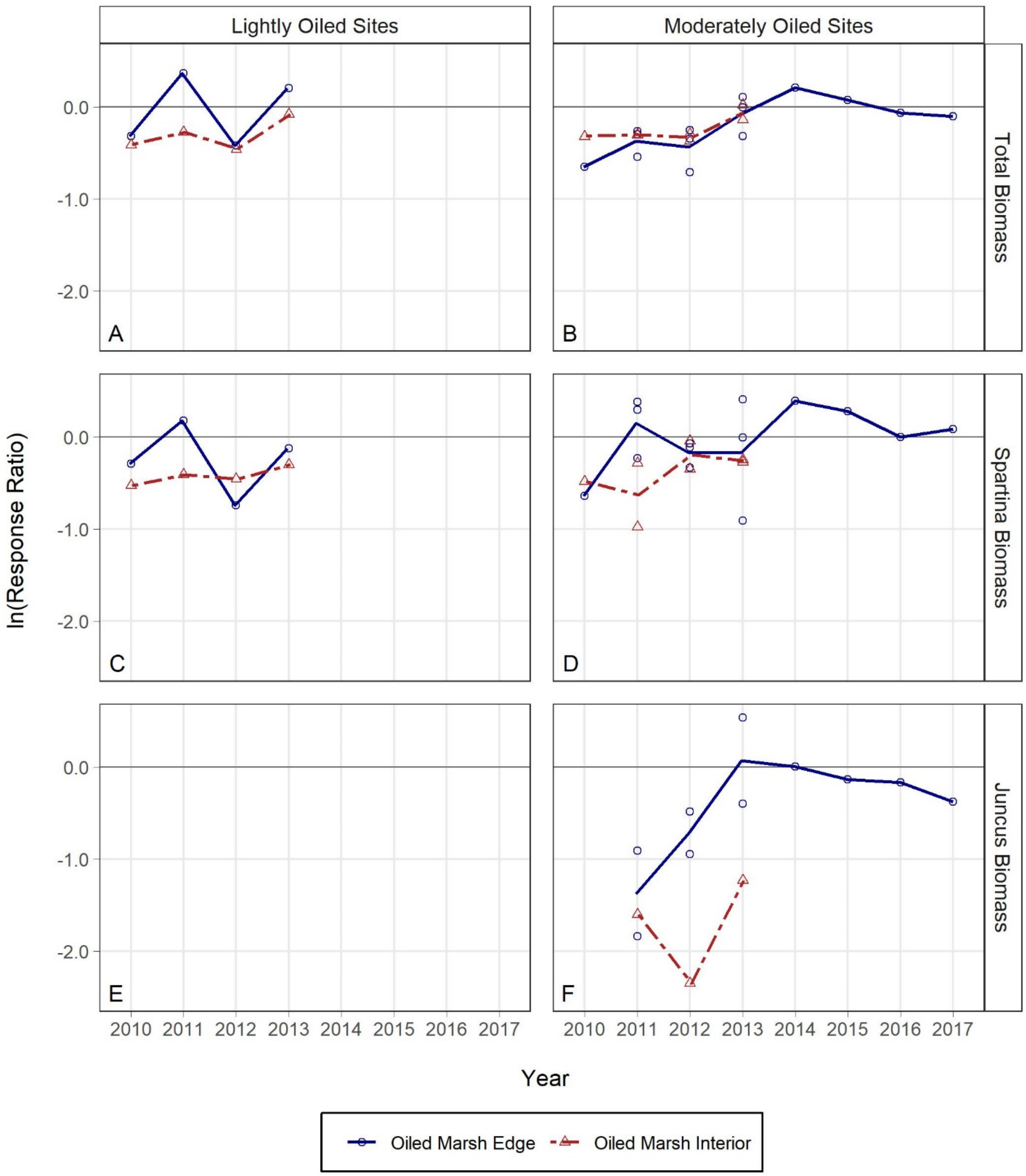

Appendix S2: Figure S4. Response ratios (ln[RR]) (oiled/reference) for aboveground biomass (g m^{-2}) by marsh zone and year for: (A) total aboveground biomass for lightly oiled sites, (B) total aboveground biomass for moderately oiled sites, (C) *Spartina alterniflora* aboveground

biomass for lightly oiled sites, (D) *Spartina alterniflora* aboveground biomass for moderately oiled sites, (E) *Juncus roemerianus* aboveground biomass for lightly oiled sites (no data), and (F) *Juncus roemerianus* aboveground biomass for moderately oiled sites. Lines connect mean annual ln(RR) values by marsh zone across years. ln(RR) values from each contributing source are plotted as open symbols. ln(RR) values less than zero indicate reductions in metrics for oiled sites compared to reference (= impacts). As a guide to interpreting degree of effects, ln(RR) = -0.7 is a ~50% reduction for oiled sites relative to reference, ln(RR) = -2.3 is a ~90% reduction, and ln(RR) = -4.6 is a ~99% reduction.

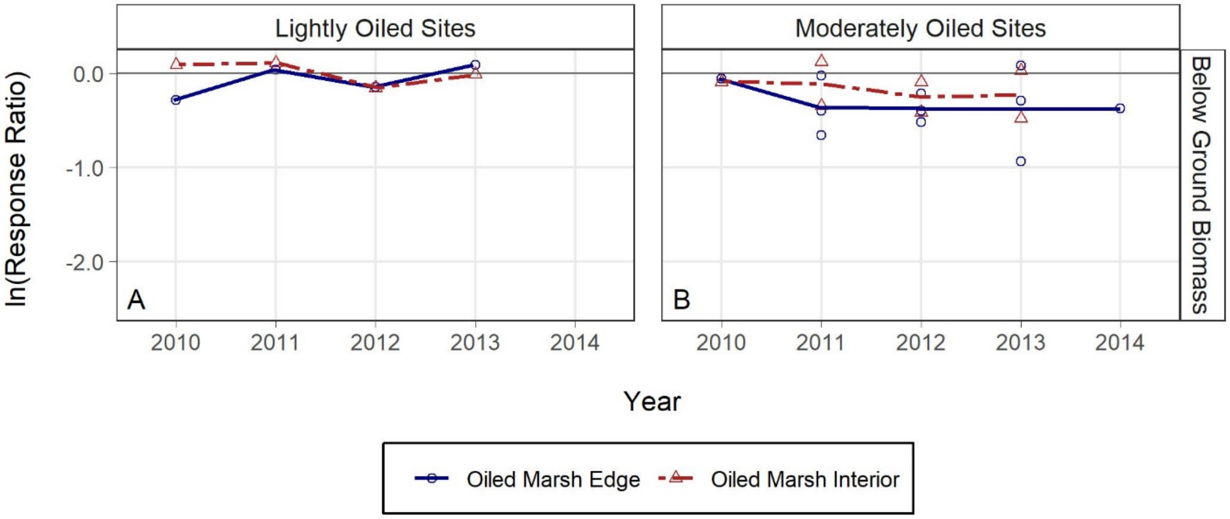

Appendix S2: Figure S5. Response ratios (ln[RR]) (oiled/reference) for belowground biomass (g m^{-2}) by marsh zone and year for: (A) lightly oiled sites, (B) moderately oiled sites. Lines connect mean annual ln(RR) values by marsh zone across years. ln(RR) values from each contributing source are plotted as open symbols. ln(RR) values less than zero indicate reductions in metrics for oiled sites compared to reference (= impacts). As a guide to interpreting degree of effects, ln(RR) = -0.7 is a ~50% reduction for oiled sites relative to reference, ln(RR) = -2.3 is a ~90% reduction, and ln(RR) = -4.6 is a ~99% reduction.
